# Breaking the 1,2-HOPO barrier with a cyclen backbone for more efficient sensitization of Eu(iii) luminescence and unprecedented two-photon excitation properties†
†Electronic supplementary information (ESI) available. See DOI: 10.1039/c9sc00244h


**DOI:** 10.1039/c9sc00244h

**Published:** 2019-03-28

**Authors:** Lixiong Dai, Wai-Sum Lo, Yanjuan Gu, Qingwu Xiong, Ka-Leung Wong, Wai-Ming Kwok, Wing-Tak Wong, Ga-Lai Law

**Affiliations:** a The Hong Kong Polytechnic University Shenzhen Research Institute , Shenzhen , PR China . Email: ga-lai.law@polyu.edu.hk ; Email: w.t.wong@polyu.edu.hk; b State Key Laboratory of Chemical Biology and Drug Discovery , Department of Applied Biology and Chemical Technology , The Hong Kong Polytechnic University , Hung Hom , Kowloon , Hong Kong SAR , PR China; c Department of Chemistry , Hong Kong Baptist University , Kowloon Tong , Hong Kong SAR , PR China

## Abstract

A cyclen backbone was utilized to study the effect of backbone rigidity on Eu(iii) luminescence sensitization using a 1,2-HOPO derivative and 2-thenoyltrifluoroacetonate (TTA) as chromophores. The restriction of molecular movement of **Eu-Cy-HOPO** brought about by the increased rigidity provided a tightly packed coordination environment for the octadentate Eu(iii) center which resulted in the highest overall quantum yield (30.2%) and sensitization efficiency (64.6%) among 1,2-HOPO sensitized Eu(iii) complexes. **Eu-Cy-HOPO** is also the first 1,2-HOPO-based lanthanide complex to emit Eu(iii) luminescence under two-photon excitation.

## Abstract 2

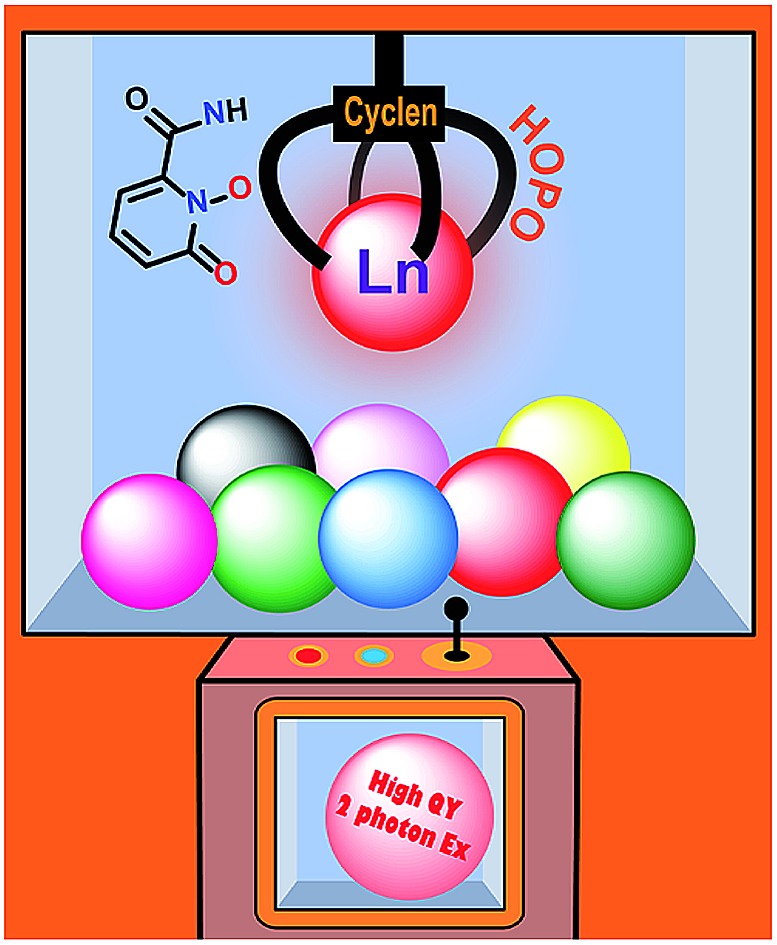
Breaking the barrier of 1,2 HOPO complexes with extremely emissive **Eu-Cy-HOPO** (overall quantum yield –30.2%) that displays two photon properties.

## Introduction

Trivalent lanthanide (Ln(iii)) ions exhibit unique luminescence properties, such as fingerprint spectral profiles, sharp emission bands and long luminescence lifetimes and thus are widely considered as potential replacements of organic chromophores and transition metal complexes especially in biological applications in which autofluorescence from biological entities often interferes with fluorescent signals due to their similar emission lifetimes and/or emission profile.^1^–^3^ However, optimizing lanthanide luminescence requires delicate molecular design to fulfill vital elements during the sensitization process. The f–f transitions of Ln(iii) ions are Laporte forbidden and therefore have intrinsically low absorption coefficients (*ε* ≈ 1–10 M^–1^ cm^–1^). Indirect excitation (sensitization) of Ln(iii) is achieved by an excited energy transfer process from a light-harvesting chromophore and the energy transfer efficiency is principally governed by two main energy transfer mechanisms dependent on the chromophore-Ln(iii) distance. The Förster mechanism is a ‘through space’ interaction that requires the donor's emission spectrum to overlap with the acceptor's excitation spectrum with a *r*^–6^ distance-dependence.^4^,^5^ Alternatively, the Dexter mechanism requires a physical orbital overlap between the donor and acceptor for a concerted electron exchange and is dependent on e^(–2*r*/*L*)^.^5^ Secondly, as the 4f electrons of Ln(iii) are well-shielded, interaction between Ln(iii) ions and ligands is mainly ionic, resulting in flexible coordination numbers from 8–12 depending on steric factors. It has therefore become prevalent to design multidentate chromophoric ligands to obtain good luminescence properties.

In 2006, Raymond's group extended Picard's work^6^ on using a bidentate 1,2-hydroxypyridinonate (1,2-HOPO) chromophore to sensitize Ln(iii) by modifying it into a tetradentate ligand consisting of two 6-amide derivatives of 1,2-HOPO to form an ML_2_ complex and dramatically improved the luminescence properties of the Eu(iii) complex: from *Φ* = 0.3% of Picard's group to 21.5%.^7^ Then, they designed the first example of an octadentate ligand with four units of the 1,2-HOPO derivative connected through an *N*,*N*,*N*′,*N*′-tetrakis-(2-aminoethyl)-ethane-1,2-diamine (H(2,2)) to form an ML complex with improved aqueous stability but exhibited weak luminescence (*Φ* = 3.6%) due to the presence of one water molecule in the inner coordination sphere of Eu(iii).^8^ Replacing the branched tetrapodal skeleton of H(2,2) with a linear spermine-based (3,4,3-LI) offered sufficient protection of the Eu(iii) center from water molecules coordination and directly resulted in an increased radiative decay rate and decreased non-radiative decay rate; however, the quantum yield was only 7.0% as the linear backbone contributed to poorer sensitization.^9^ Later, they found out that isolating the same (3,4,3-LI) Eu(iii) complex prior to luminescence measurements would allow the 1,2-HOPO derivative units to fully coordinate with Eu(iii) and lead to a slower non-radiative decay rate and higher sensitization efficiency, achieving a quantum yield of 15.6%.^10^ In 2015, a systematic study on derivatives based on the H(2,2) skeleton was carried out on investigating how the change of central chain length and the length between two bridgehead tertiary nitrogen atoms would affect the photophysical properties of Eu(iii).^11^ The authors concluded that with a shorter length, the steric constraints would lead to coordination of a water molecule whereas a longer length would give highly luminescent complexes (*Φ* = 19.6%). Most recently, the highest quantum yield of an Eu(iii) complex sensitized by the 6-amide 1,2-HOPO derivative was obtained (*Φ* = 23.9%) with a tetradentate ligand with two methylene groups between the two chelating chromophores, and such a geometry allows better wrapping of the Eu(iii), resulting in better sensitization efficiency.^16^ Nonetheless, a systematic relationship between the coordination geometry and photophysical properties remains elusive despite more than a decade's thorough work on a selected system. Table 1 selectively summarizes the work of Raymond's group.

**Table 1:** Summary of literature data of Eu(iii) complexes sensitized by 1,2-HOPO derivatives done by Raymond's group^*a*^

Compound	*ε* (M^–1^ cm^–1^)	*τ*_H_2_O_ (ms)	*q*	*Φ* Ln L	*Φ* Ln Ln	*η*_sens_	Ref.
[Eu(5LIO-1,2-HOPO)_2_]^–^	19 250@333 nm	0.727	0	21.5%	43.0%	49.0%	7
[Sm(5LIO-1,2-HOPO)_2_]^–^	19 200@331 nm	0.013	—	0.44%	—	—	12
Eu(5LIN^Me^-1,2,-HOPO)_2_	18 750@332 nm	0.728	0	0.173	44.2%	39.1%	8
[Eu(H(2,2)-1,2-HOPO)]^–^	18 200@341 nm	0.48	1	3.6%	17.8%	20.2%	13
[Eu(*o*-Phen-1,2-HOPO)_2_]^–^	21 020@342 nm	0.536	0	6.2%	36.5%	17.0%	14
[Eu(5LI-1,2-HOPO)_2_]^–^	18 800@331 nm	0.737	0	20.7%	49.0%	42.0%	12
[Eu(5LI-1,2-HOPO)_2_]^–^	19 400@331 nm	0.011	—	0.44%	—	—	12
[Eu(3,4,3-LI(1,2,-HOPO))]^–^ (*in situ*)	17 700@315 nm	0.805	0	0.7%	43.2%	16.2%	9
[Sm(3,4,3-LI(1,2,-HOPO))]^–^ (*in situ*)	17 850@316 nm	0.017	—	0.2%	—	—	15
[Eu(3,4,3-LI(1,2,-HOPO))]^–^ (isolated)	—	0.814	0	15.6%	46.9%	39.7%	10
[Sm(3,4,3-LI(1,2,-HOPO))]^–^ (isolated)	—	0.019	—	0.41%	—	—	10
[Eu(H(17O5,2)-1,2-HOPO)]^–^	15 000@336 nm	0.704	0	19.6%	52.2%	37.5%	11
[Eu(2LI-1,2-HOPO)_2_]^–^	21 600@338 nm	0.578	0	23.9%	42.0%	51.0%	16
[Sm(2LI-1,2-HOPO)_2_]^–^	21 600@334 nm	0.017	0	0.4%	—	—	16

^*a*^All measurements were done at pH 7.4.

1,4,7,10-Tetraazacyclododecane (cyclen)-based chelators are vastly common amongst Ln(iii) and transition metals for various applications, especially the carboxylate derivative DO3A (and its derivatives) which gives octadentate complexes with exceptional stability.^17^–^19^ In this work, we intend to utilize the 12-memebered ring as a macrocyclic backbone and investigate how it would influence the molecular arrangement and hence the luminescence properties of the complexes compared to tetrapodal and linear backbones. We expect the relatively rigid cyclen ring would restrict the movement of the tetradentate 1,2-HOPO units^20^ and reduce the rate of non-radiative deactivation while simultaneously increasing the energy transfer efficiency by limiting the average Eu(iii)-1,2-HOPO separation. To further confirm the effect of the macrocyclic backbone, we also designed an analogous chelate with 2-thenoylfluoroacetonate (TTA), a known efficient sensitizer for Eu(iii) luminescence,^21^ as comparison.

While 1,2-HOPO-based and cyclen-based Ln(iii) complexes are often water-soluble, the application of luminescent Ln(iii) complexes in a biological context, in general, is often hindered by the high-energy, tissue-damaging energy required for exciting the chromophore during antenna effect. The invention of femtosecond-pulsed laser sources has made multi-photon absorption – a non-linear optical process in which two or more photons with a combined amount of energy equal to the Δ*E* of a single-photon absorption process are absorbed almost simultaneously by a molecule affording a convenient solution by significantly shifting the excitation wavelength near or beyond the red region.^22^,^23^ However, as the selection rules for single-photon, two-photon and three-photon excitation are different, chromophores with a high *ε* does not guarantee a high two-photon absorption cross section (*σ*_2_). Highly absorptive dyes such as fluorescein and rhodamine 6G, with absorption maxima at *ca.* 500 nm and 530 nm respectively, have *δ* values of 8.0 and 9.2 GM (1 GM = 10^–40^ cm^4^ s photon^–1^) at 950 nm.^24^ Following a systematic study, Albota *et al.* suggested a design rationale for chromophores with a high *σ*_2_: ‘π-conjugated molecules with large changes of quadrupole moment upon excitation’^25^ and organic chromophores with *d* > 5000 GM have been gradually developed.^26^–^29^ Ln(iii) complexes containing chromophores with *σ*_2_ from 0.37 to 775 GM have also been reported.^21^,^30^,^31^ As a result, a suitable balance between electronic density gradient and water-solubility should be attained when designing the structure of chromophores for two-photon biological applications.

## Results and discussion

### Synthesis of ligands and complexes

**Scheme 1:**
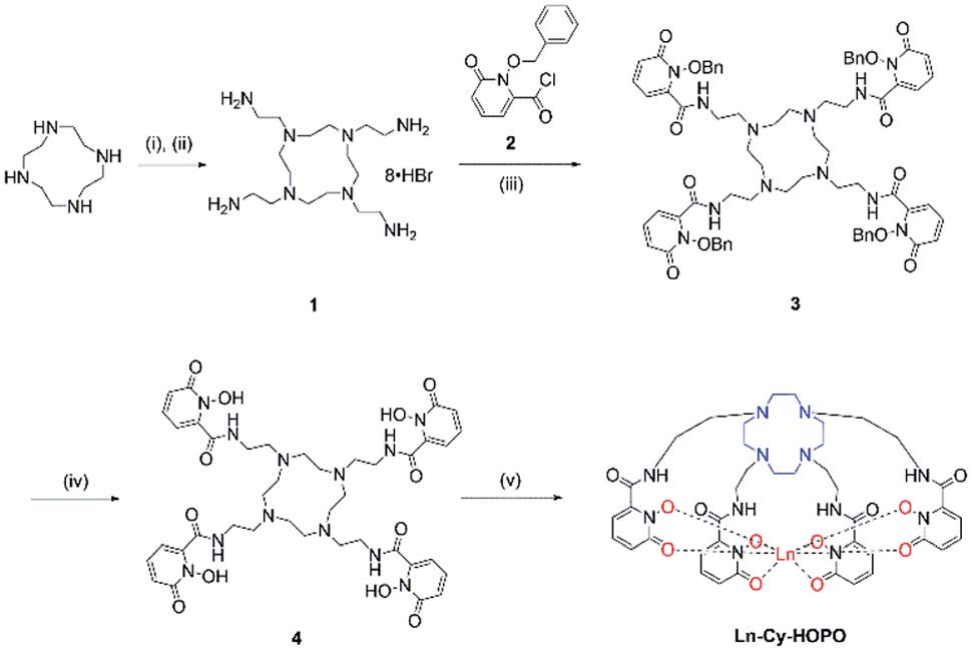
Synthetic route of **Ln-Cy-HOPO**. (i) tosylaziridine, MeCN/benzene; (ii) AcOH/HBr; (iii) DIPEA, THF; (iv) AcOH/HCl; (v) LnCl_3_·6H_2_O, pyridine, MeOH, 55 °C, 8 h.

**Fig. 1:**
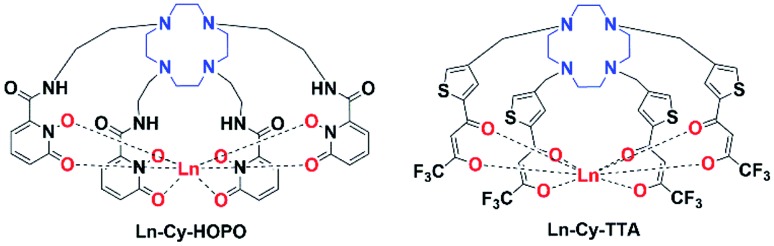
Structures of europium(iii) complexes **Ln-Cy-HOPO** and **Ln-Cy-TTA** (Ln = Eu/Sm/Gd).

The syntheses of ligand **4** is presented in Scheme 1. Originally, we tried reacting cyclen with phthalimide- or Boc-protected bromoethylamine to give a protected tetra-amine cyclen derivative, but both reaction routes gave very low yields due to the reactivity of the bromo group and side reactions from nucleophilic substitution. By using tosylaziridine in a ring-opening, zwitterion-forming reaction, the protected derivative was obtained in a good yield after recrystallization with acetonitrile and benzene. Deprotection with acetic acid and hydrobromic acid gave **1** readily for reaction with the protected 1,2-HOPO derivative to give **3**, which was purified by semi-preparative HPLC. The octadentate ligand **4** was obtained by recrystallization with methanol and diethyl ether after deprotection. Complexation with Ln(iii) trichloride hexahydrate was performed in methanol in the presence of pyridine at 55 °C for 8 hours (Fig. 1).

**Scheme 2:**
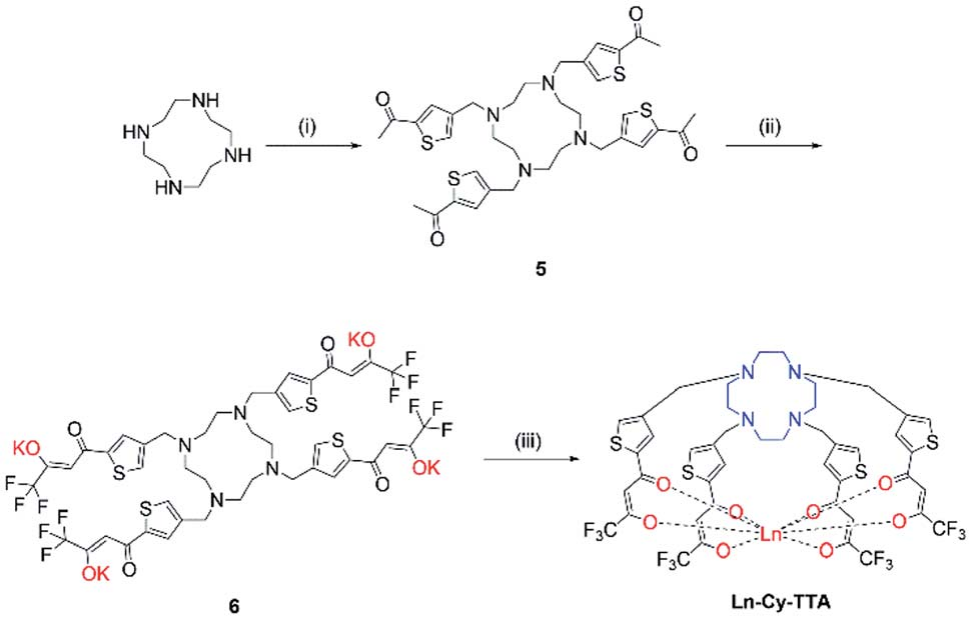
Synthetic route of **Ln-Cy-TTA**. (i) 4-Chloromethyl-2-acetylthiophene, K_2_CO_3_, ACN, RT, 48 h; (ii) ethyl trifluoroacetate, KHMDS, –78 °C, RT; (iii) LnCl_3_·6H_2_O, MeOH, 60 °C, 12 h.

Chloromethylation of 2-acetylthiophene was performed successfully with aluminum chloride as Lewis acid and strict control over the reaction time and stoichiometry and was subsequently reacted with cyclen to give **5**. The substitution reaction was performed at room temperature for two days since a lot of side products – reaction of ketone with amine and acetylthiophene should be well-controlled, too, to avoid over- alkylation. Compound **6** was obtained by reacting **5** with ethyl trifluoroacetate with potassium bis(trimethylsilyl)amide and complexation was carried out with Eu(iii) trichloride hexahydrate in methanol at 60 °C overnight (Scheme 2).

### Photophysical properties

#### UV-vis absorption spectroscopy

**Fig. 2:**
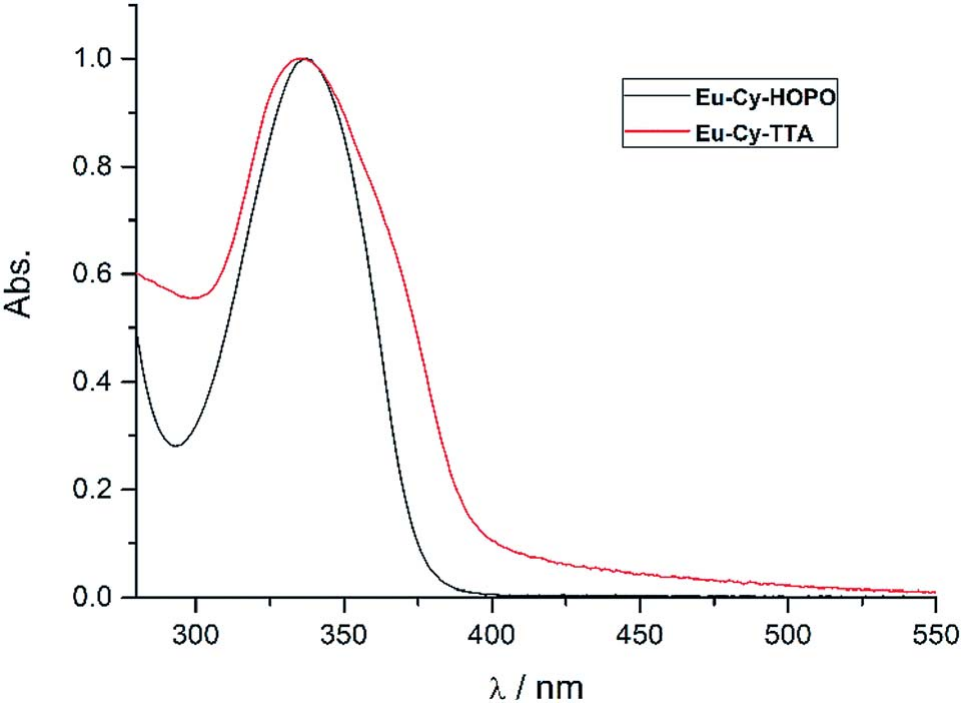
Normalized UV-vis absorption spectra of **Eu-Cy-HOPO** and **Eu-Cy-TTA**.

Fig. 2 shows the UV-vis absorption properties of **Eu-Cy-HOPO** and **Eu-Cy-TTA** in water (pH 5.5) and aqueous solution (3% DMSO) respectively. Both absorption spectra only show one absorption band with maxima at 337 nm (*ε*_350 nm_ = 12 100 M^–1^ cm^–1^) and 336 nm (*ε*_350 nm_ = 22 690 M^–1^ cm^–1^), assigned as the π–π* transitions of the chromophores. The molar absorption coefficients of are lower than values of reported 1,2-HOPO- and TTA-based compounds, due to the crowdedness between the chromophores brought about by the rigid cyclen backbone.

#### Luminescence properties of **Eu-Cy-HOPO** and **Eu-Cy-TTA**

**Fig. 3:**
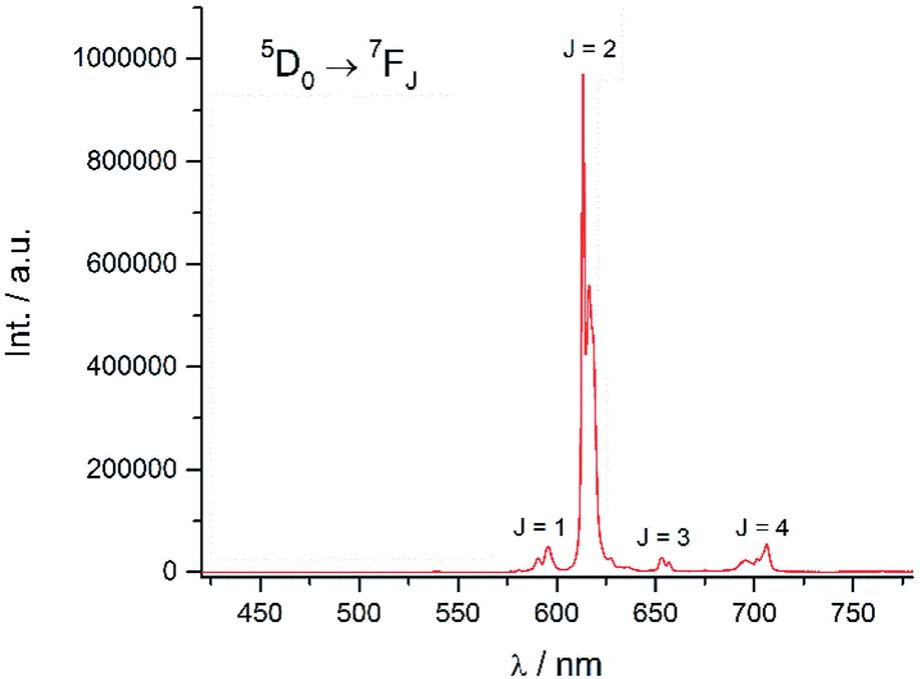
Emission spectrum of **Eu-Cy-HOPO** in water (*λ*_ex_ = 350 nm).

**Fig. 4:**
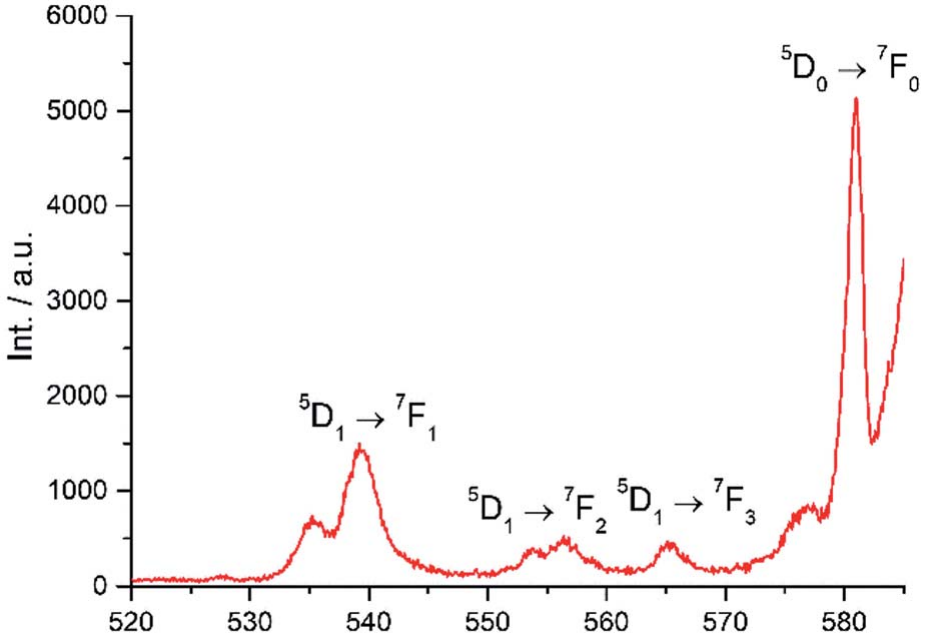
Higher magnification of partial emission spectrum of **Eu-Cy-HOPO** in Fig. 3.

Excitation at 350 nm resulted in the characteristic Eu(iii) emission profile with the ^5^D_0_ → ^7^F_J_ (*J* = 1–4) transitions clearly observed (Fig. 3). Fig. 4 offers a higher magnification into the ^5^D_0_ → ^7^F_0_ transition, which is often very weak in due to its forbidden nature, as well as the ^5^D_1_ → ^7^F_J_ transitions, indicating the involvement of the higher excited state in sensitization.

**Fig. 5:**
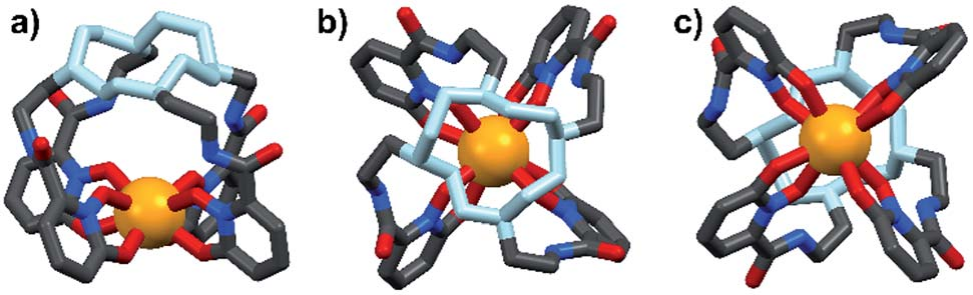
Optimized structure of **Eu-Cy-HOPO**. View from side (a); above cyclen backbone (b); view from below Eu(iii) center (c).

Energy transfer from the 1,2-HOPO unit is efficient as residual ligand fluorescence was not observed. The high intensity of the ^5^D_0_ → ^7^F_2_ hypersensitive transition relative to the other transitions, quantified by an asymmetry ratio of 14, reveals a large extent of deviation from a centrosymmetric geometry of the Eu(iii) center,^32^ corroborating with the narrow octadentate structure optimized using the RM1 model by the LUMPAC software package (Fig. 5).^33^,^34^

The luminescence lifetime of the ^5^D_0_ → ^7^F_2_ transition was measured to be a respectable 0.784 ms. The number of coordinated water molecule in the inner coordination sphere (*q*) of Eu(iii) was determined to be 0 using both Parker's^35^ and Horrocks'^36^ equation respectively, which is similar to the octadentate complexes of Raymond's group. The luminescence lifetime was also measured in methanol and methanol-d_4_ and the number of coordinated methanol molecule (*m*) is determined to be 0.^37^ An overall quantum yield of 30.2% was measured relative to quinine sulfate; this value is the highest amongst Eu(iii) complexes sensitized by a 1,2-HOPO chromophore thus far. The sensitization efficiency in water was calculated to be 64.6%, which is noticeably higher compared to the ceiling of 50% of Raymond's group in 0.1 M TRIS buffer (Table 1). These parameters indicated efficient luminescence sensitization which is attributed to two factors from our molecular design: (1) sufficient protection by the four 1,2-HOPO units prevented the coordination of solvent molecules which quenches the excited energy of Eu(iii) by vibrational overtones of O–H oscillators^35^ and; (2) rigid macrocyclic backbone restricting the movement of the 1,2-HOPO unit and maintaining a close distance between the chromophore and Eu(iii) since energy transfer mechanisms are highly distance-dependent.

**Fig. 6:**
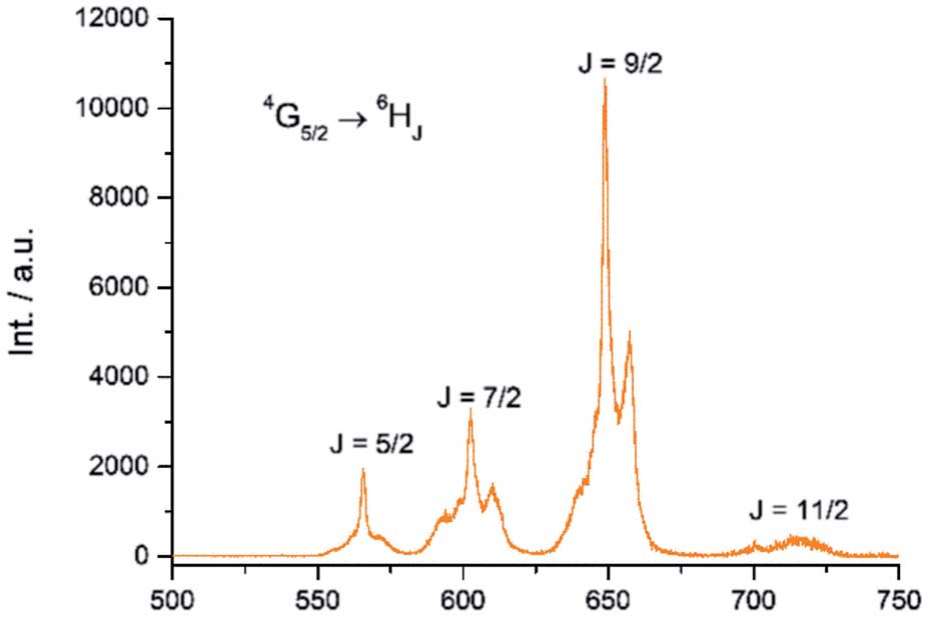
Emission spectrum of **Sm-Cy-HOPO** in water (*λ*_ex_ = 350 nm).

**Fig. 7:**
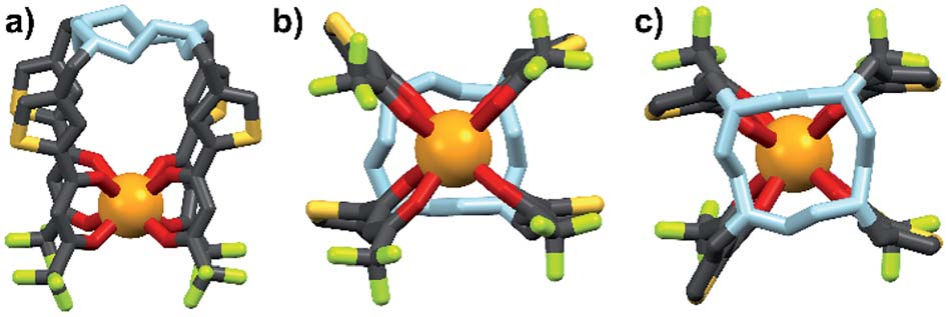
Optimized structure of **Eu-Cy-TTA**. View from side (a); view from above cyclen backbone (b); view from below Eu(iii) center (c).

**Fig. 8:**
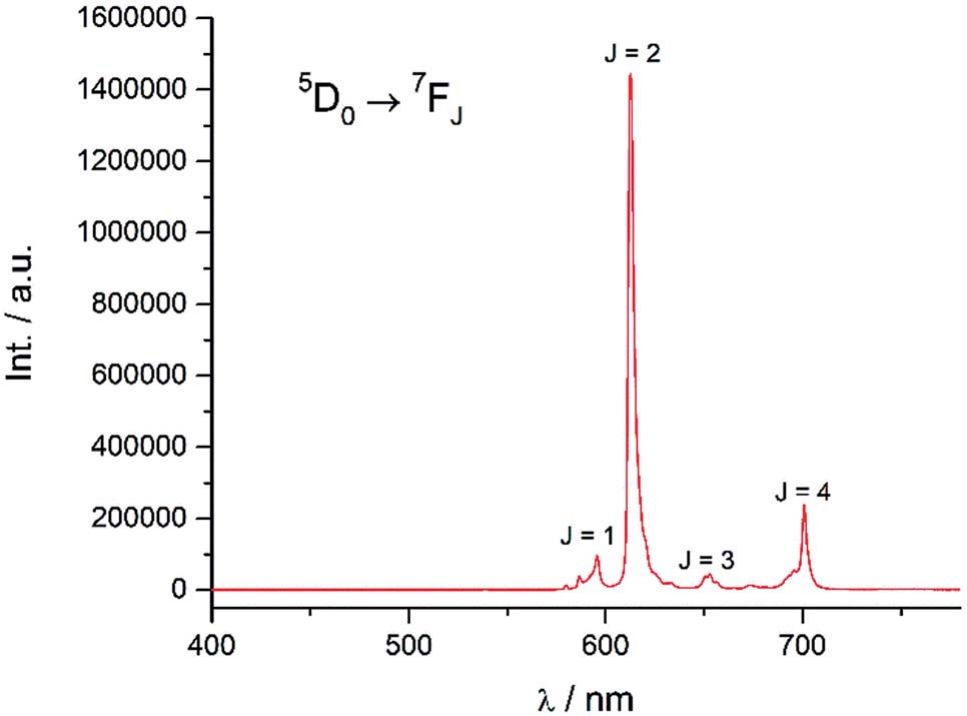
Emission spectrum of **Eu-Cy-TTA** in 3% DMSO aqueous solution (*λ*_ex_ = 350 nm).

The Sm(iii) analog, **Sm-Cy-HOPO**, was synthesized as chromophores that could sensitize Eu(iii) luminescence could often sensitize Sm(iii) luminescence as well (*vide infra*). As seen in Fig. 6, certain ^4^G_5/2_ → ^6^H_J_ transitions (*J* = 5/2–11/2) in the visible region could be observed by exciting the ligand at 350 nm. The luminescence lifetime of the most intense ^4^G_5/2_ → ^6^H_9/2_ transition was measured to be 16 μs in water and the *q* value was determined to be 0 and 0.5 by Kimura's^38^ and Hakala's^39^ equation respectively (Table 2). While it is impractical for half a water molecule to be coordinated, this value reflects that the Sm(iii) is not as well secluded from water molecules by the macrocyclic ligand as the Eu(iii) counterpart due to the Sm(iii)'s slightly larger ionic radius (Fig. S41†) and this is also supported by a larger *m* value. Furthermore, as the energy gap between the emitting state and the next lower energy level of Sm(iii) is quite small (*vide infra*), Sm(iii) complexes suffers an intrinsic disadvantage of having low luminescent quantum yields and thus the 0.4% determined for **Sm-Cy-HOPO** is not surprising. The ^5^D_0_ → ^7^F_J_ transitions (*J* = 0–4) and some of the ^5^D_1_ → ^7^F_J_ (*J* = 0–3) could be clearly observed when **Eu-Cy-TTA** was excited at 350 nm in aqueous solution (Fig. 8). Like **Eu-Cy-HOPO**, the ^5^D_0_ → ^7^F_2_ transition is more intense than other transitions, yet the coordination environment is expected to be slightly different since the asymmetry ratio is 11.5 and the splitting of the hypersensitive transition is not the same.^40^ The luminescence lifetime measured in aqueous solution (3% DMSO) was best-fitted with a bi-exponential decay (0.968 and 0.377 ms), indicating the presence of two radiatively decaying species. The shorter-lived species is believed to be due to coordinated water molecules, however, the presence of DMSO renders the calculation of *q* value inaccurate. Alternatively, the *m* value^41^,^42^ – representing the number of coordinated methanol molecules – was determined to be 2. It is also worth mentioning that **Eu-Cy-TTA** exhibited a mono-exponential decay in methanol with a much shorter lifetime (0.463 ms), suggesting the complex is more vulnerable to methanol coordination than water molecules. Nonetheless, in aqueous solution, a considerably decent overall quantum yield of 21.7% was recorded for **Eu-Cy-TTA** despite the co-existence of the hydrated species as indicated by the bi-exponential lifetime. Such an interesting observation could be explained by our proposed cage-like structure – supported by Sparkle optimization – such arrangement of chromophore creates much higher steric hindrance among the four TTA molecules around the Eu(iii) compared to the smaller 1,2-HOPO units, leading to a less tight structure than **Eu-Cy-HOPO**, thus allowing space for the infiltration of solvent molecules. An optimized structure was obtained from LUMPAC with RM1 model (Fig. 7).

**Table 2:** Photophysical data of **Ln-Cy-HOPO** and **Eu-Cy-TTA** in water (pH 5.5) and methanol^*a*^

	**Eu-Cy-HOPO**	**Sm-Cy-HOPO**	**Eu-Cy-TTA**
*ε* (M^–1^ cm^–1^)	12 110@337 nm	15 360@337 nm	22 690@336 nm
*τ*_H_2_O_ (ms)	0.784	0.018	0.968, 0.377
*τ*_D_2_O_ (ms)	1.06	0.113	—
*q*	0	0.5	—
*Φ* Ln L	30.2%	0.4%	21.7%
*τ*_MeOH_ (ms)	0.825	0.025	0.463
*τ*_MeOD_ (ms)	0.962	0.104	0.876
*m* 41,42	0	1.5	2

^*a*^Estimated error in *τ* and *Φ* are 10% and 15% respectively.

The sensitization efficiency was calculated by eqn (1)–(3) (Table 3). The intrinsic quantum yield (*Φ*LnLn) for **Eu-Cy-HOPO** was calculated to be 46.8%, in agreement with literature values whereas that of **Eu-Cy-TTA** is higher at 52.9%. Nonetheless, the overall quantum yield of the latter is indeed lower per the above data, resulting in a lower sensitization efficiency (*η*_sens_). The intrinsic quantum yield is defined as the quantum yield obtained from direction 4f–4f excitation whereas the overall quantum yield takes the sensitization process into account. In other words, *η*_sens_ is a parameter to evaluate the extent of excited energy lost prior to reaching the Eu(iii)'s accepting state; quenching of the Eu(iii)'s excited state, such as by overtones of O–H oscillators, is irrelevant to the sensitization. The lower *η*_sens_ of **Eu-Cy-TTA** is attributed to the steric demand of the larger TTA molecule, resulting in a longer Eu(iii)-chromophore distance (6.1 Å of **Eu-Cy-HOPO***vs.* 7.1 Å of **Eu-Cy-TTA** as measured from their optimized structures) and less efficient energy transfer *via* distance-dependent energy transfer mechanisms, as indicated by the slower rate of radiative deactivation (*τ*_rad_).

**Table 3:** Calculated Eu(iii) parameters of **Eu-Cy-HOPO** and **Eu-Cy-TTA** in water and aqueous solution respectively

	**Eu-Cy-HOPO**	**Eu-Cy-TTA**
*Φ* Ln L	30.2%	21.7%
*τ*_rad_ (ms)	1.675	1.830
*k*_rad_ (s^–1^)	597	546
*k*_nr_ (s^–1^)	678	487
*Φ* Ln Ln	46.8%	52.9%
*η*_sens_	64.6%	41.1%

#### Energy transfer pathway

**Fig. 9:**
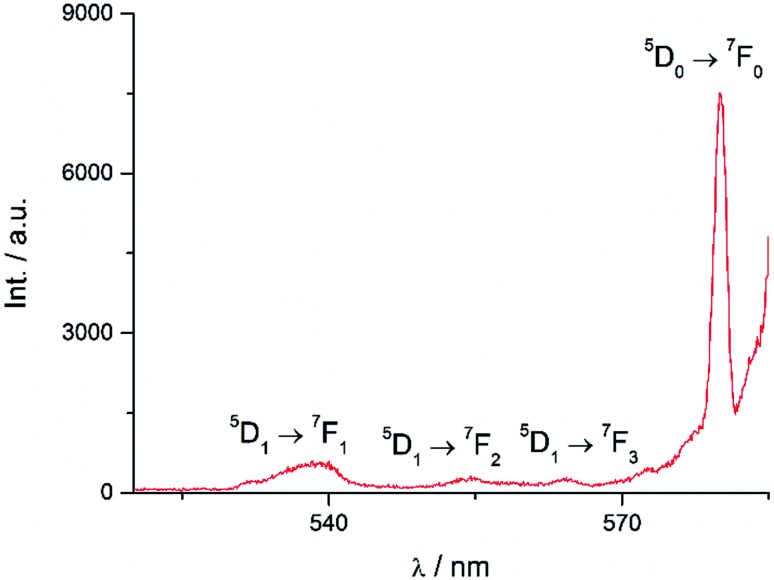
Magnification of partial emission spectrum of **Eu-Cy-TTA** in Fig. 8.

**Fig. 10:**
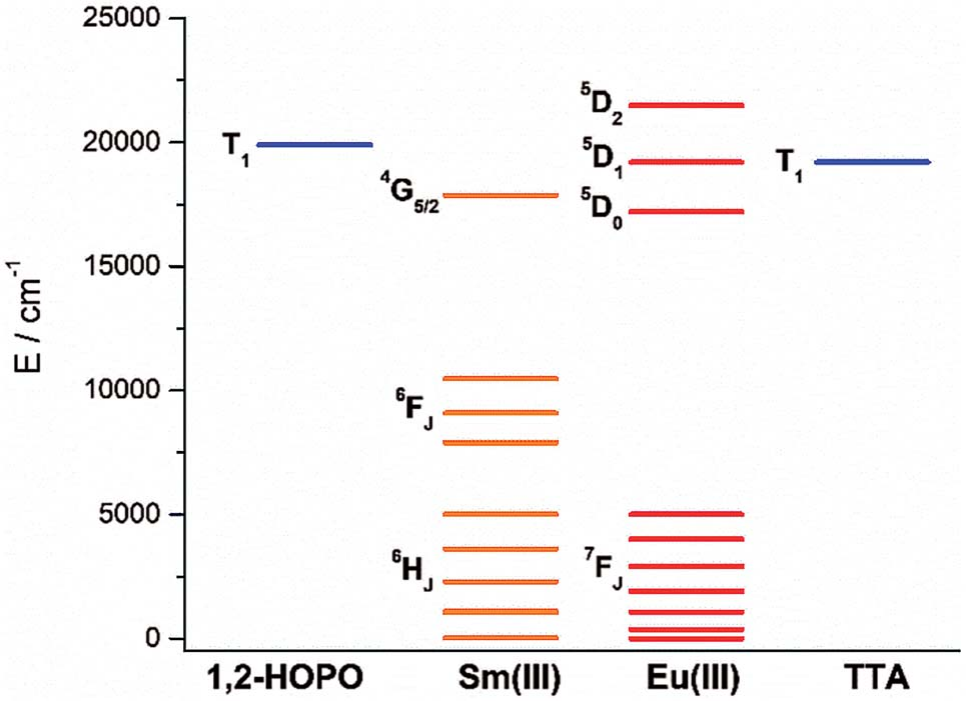
Energy level diagram depicting the energy levels of Eu(iii), Sm(iii) and the triplet states of 1,2-HOPO and TTA determined from **Gd-Cy-HOPO** and **Gd-Cy-TTA**.

To study the antenna effect, the Gd(iii) counterpart was synthesized to probe the triplet state of the chelated chromophore. Since the 4f electrons are very well-shielded, the ionic radii of Gd(iii) and Eu(iii) are very similar and hence it is commonly accepted that the coordination environments are comparable. Furthermore, the excited states of Gd(iii) are situated beyond 30 000 cm^–1^,^43^ so energy transfer is often impractical. At low temperature (77 K), reverse intersystem crossing is hindered, and the excited energy would have a higher tendency to relax from the triplet excited state to give phosphoresce. At room temperature, the emission spectrum of **Gd-Cy-HOPO** showed very weak ligand fluorescence with two emission maxima at 408 and 434 nm. After cooling **Gd-Cy-HOPO** at 77 K, a new, broad emission band with peak maximum at 503 nm was observed, and the emission band is assigned as ligand phosphorescence since the emission lifetime was determined to be 6.87 ms. For **Gd-Cy-TTA**, there was negligible emission at two band maxima at 510 nm and 534 nm appeared with biexponential lifetimes of 1.4 ms and 6.4 μs and 1.2 ms and 7.4 μs respectively. The triplet excited state of 1,2-HOPO is determined to be at *ca.* 19 900 cm^–1^ and that of TTA, taken as average of the two peak maxima, is *ca.* 19 200 cm^–1^ (Fig. S4 and S6†). The ^5^D_0_ accepting state of Eu(iii) is at *ca.* 17 200 cm^–1^,^44^ and the energy gap between the respective triplet states and the accepting state(s) falls within the ideal 2500 to 4000 cm^–1^ range for efficient energy transfer while preventing thermally-promoted back energy transfer.^45^ The higher excited state ^5^D_1_, at *ca.* 19 200 cm^–1^, despite its proximity with the triplet energy levels, is also involved as shown in Fig. 4 and 9. Furthermore, the exceptionally long phosphorescence lifetime – implying a low non-radiative deactivation rate of the excited triplet state – provides a stable and long-lived excited state for energy transfer to take place, resulting in efficient luminescence sensitization. On the other hand, the ^4^G_5/2_ accepting state of Sm(iii) is located at *ca.* 17 860 cm^–1^,^46^ and is therefore expected to be the recipient of the excited energies from HOPO and TTA. While the energy gap between the emitting state and the next lower energy state of Sm(iii) (Δ*E*_(^4^G_5/2_→^6^F_11/2_)_ = *ca.* 7500 cm^–1^) does not resonate with oscillator overtones, a closer examination of the next energy level (Δ*E*_(^4^G_5/2_→^6^F_9/2_)_ = *ca.* 8700 cm^–1^) reveals a close match between the second C–H overtone (*ca.* 8700 cm^–1^), leading to efficient non-radiative deactivation (Fig. 10). This result is consistent with Doffek *et al.*'s finding regarding how the smallest energy gap is not ‘universally relevant’, especially in Sm(iii) contexts.^47^ Consequently, luminescent quantum yields of organo-Sm(iii) complexes are generally expected to be low due to the abundant C–H oscillators in proximity, and this also explains why only the sensitization barrier of Eu(iii) luminescence could be broken by a change to the cyclen backbone but not Sm(iii)'s, with the quantum yield of **Sm-Cy-HOPO** (0.4%) the same as those reported by Raymond's group.

#### Two-photon absorption and excitation

**Fig. 11:**
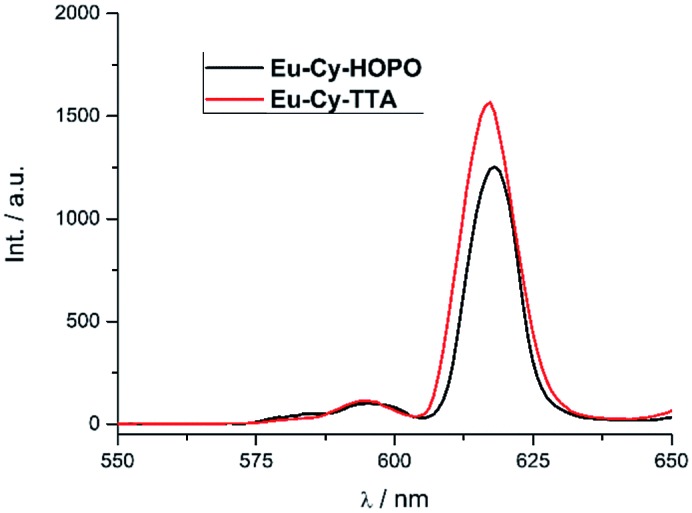
Emission spectra of **Eu-Cy-HOPO** and **Eu-Cy-TTA** under two-photon excitation in DMSO (*λ*_ex_ = 700 nm).

Under two-photon excitation at 700 nm with an ultrafast Ti:Sapphire laser, Eu(iii) luminescence spectra were recorded for **Eu-Cy-HOPO** and **Eu-Cy-TTA** in DMSO (Fig. 11). The emission profile is typical of Eu(iii) and the intense ^5^D_0_ → ^7^F_2_ transitions relative to the ^5^D_0_ → ^7^F_1_ transition resemble those in Fig. 3 and 8, suggesting the same emitting species compared to single-photon excitation. The two-photon excitation mode was confirmed by the dependence of luminescence intensity on incident power (Fig. S35 and S37†). The two-photon absorption cross sections (*σ*_2_) were determined against fluorescein chromophores are not structurally constructed with a large quadrupole moment upon photo-excitation, *i.e.* the expected to play any role in the two-photon absorption process as it is spatially distant from and has minimal influence on the electronic environment of both the chromophore and the Eu(iii). Nevertheless, the unprecedented observation of Eu(iii) luminescence *via* two-photon excitation of a 1,2-HOPO-based chromophore is an encouraging result to develop Eu-Cy-HOPO for two-photon optical microscopy given its excellent water-solubility.

#### MTT assay and *in vitro* imaging

**Fig. 12:**
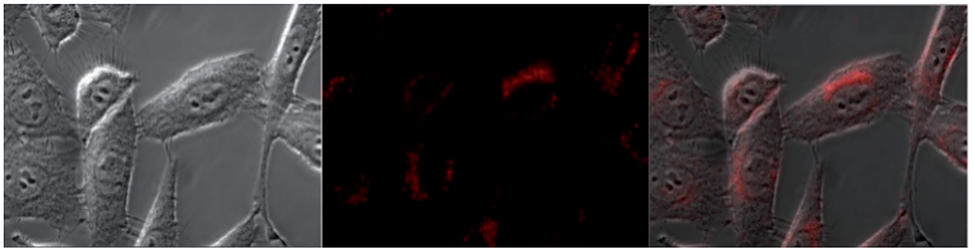
Bright field (left) and fluorescent microscopy image (middle) and overlaid image of **Eu-Cy-HOPO** (2 μM) in HeLa cells after 3 hours of incubation (*λ*_ex_ = 380 nm, BP filter 550–650 nm).

**Fig. 13:**
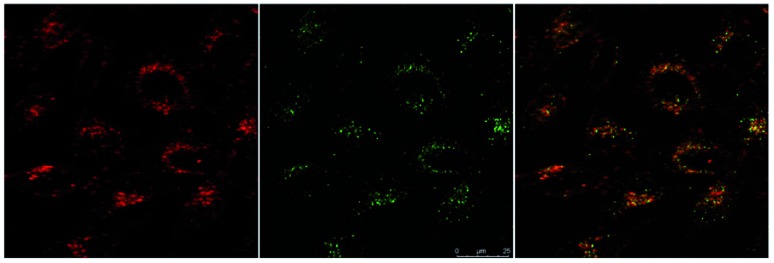
Multi-photon confocal microscopy images of **Eu-Cy-HOPO** (4 μM) after 3 hours of incubation (*λ*_ex_ = 760 nm) (left), LysoTracker® (*λ*_ex_ = 488 nm) (middle) and overlaid image (right).

**Eu-Cy-HOPO** exhibited low cytotoxicity as its IC_50_ value was determined to be 600 μM by MTT assay in HeLa cells (Fig. S39†), and its cellular uptake behavior was evaluated by fluorescent microscopy and multiphoton confocal microscopy in HeLa cells, too. Fig. 12 shows the uptake of **Eu-Cy-HOPO** by HeLa cells after 3 hours of incubation as indicated by the red luminescence under 380 nm excitation. Multiphoton excitation at 760 nm by a femtosecond pulsed laser under a confocal microscope also gave red luminescence, and the localization of **Eu-Cy-HOPO** in the lysosomes was confirmed by co-staining with LysoTracker® (Fig. 13). Due to the much lower luminescent quantum yield of **Sm-Cy-HOPO**, a higher incubation concentration (40 μM) and longer incubation time (24 hours) was required for orange-red luminescence to be observed *via* multiphoton excitation at 780 nm (Fig. S40†). On the other hand, precipitation was observed for **Eu-Cy-TTA** in aqueous solutions at concentrations used for *in vitro* studies, therefore no studies were performed.

## Conclusions

In this work, a 1,2-HOPO derivative was incorporated into a rigid cyclen backbone and the overall quantum yield of the resulting Eu(iii) complex **Eu-Cy-HOPO** was determined to be 30.2%, with a sensitization efficiency of 64.6%, both the highest thus far amongst 1,2-HOPO-based Eu(iii) complexes. The rigidity of the backbone restricts the movement of the pendant chromophores to a higher extent than the linear and branched backbones reported in literature, hence leading to less non-radiative energy loss and a closer Eu(iii)-chromophore distance for more efficient energy transfer. A TTA analog, **Eu-Cy-TTA** also gave decent luminescent properties as a result, but the steric hindrance among the TTA units allowed space for solvent molecules to exploit and penetrate the inner coordination sphere of the Eu(iii). Eu(iii) luminescence was also unprecedentedly observed under two-photon excitation of the 1,2-HOPO-based chromophore by a femtosecond laser (and in two-photon confocal microscopy), displaying emission profile and lifetimes near-identical to the single-photon excitation process. In addition, co-staining experiments with LysoTracker® confirmed the localization of **Eu-Cy-HOPO** in lysosomes *in vitro*.

## Experimental

### Materials and methods

Unless noted otherwise, all chemicals were of reagent grade and were purchased from Sigma-Aldrich or Acros Organics and used without further purification. Moisture-sensitive synthetic procedures were performed under a nitrogen atmosphere using standard Schlenk techniques. Davisil silica gel (40–63 μm) was obtained from Grace Davison. Analytical reagent grade solvents were used, and acetonitrile was dried with sodium hydride and distilled under nitrogen. ^1^H, ^13^C and ^19^F NMR spectra were recorded on a Bruker Ultrashield 400 Plus NMR spectrometer (at 400 MHz, 100 MHz and 376 MHz respectively) or a Bruker Ultrashield 600 Plus NMR spectrometer (at 600 MHz and 150 MHz respectively). The ^1^H and ^13^C NMR chemical shifts were referenced to solvent residual peaks. Mass spectra, reported as *m*/*z*, was obtained either on a Micromass Q-TOF 2 mass spectrometer or on an Agilent Technologies 6540 UHD Accurate-Mass Q-TOF LC/MS system or on a Bruker UltrafleXtreme Matrix Assisted Laser Ionization (MALDI) Mass Spectrometer. Analytical high performance liquid chromatography (HPLC) was performed on Waters 1525 series apparatus with PDA detector. The method used on this system is as follows: Atlantis T3 column (4.6 × 250 mm), mobile phase of water (with 0.05% TFA) with 10% of ACN was increased to 100% ACN within 15 min, then maintained at 100% ACN for 5 min and re-equilibrated for 5 min. Reverse-phase semi-preparative purification was performed on Waters 2535 series apparatus with PDA detection and Fraction Collector III. The method used on this system is as follows: Atlantis T3 column (19 × 250 mm), mobile phase of water (with 0.05% TFA) with 30% methanol was gradient increased to 100% methanol within 20 min, then the system was re-equilibrated for 4 min. Inductively coupled plasma - optical emission spectrometry (ICP-OES) was performed on an Agilent 700 Series system, with 6 points standards (0.5–20 ppm) of Eu, Sm and Gd in 2% of HNO_3_ for the determination of metal content. Fourier Transform Infrared (FT-IR) spectra were recorded on a Nicolet iS 50 FT-IR spectrometer with a KBr pellet.

### Photophysical measurements

Milli-Q water (18.2 MΩ cm at 25 °C) was used for aqueous measurements; methanol used were of CHROMASOLV®Plus grade from Sigma-Aldrich, deuterated water and methanol used were from Cambridge Isotope; all were used without further purification. Solution samples of *ca.* 0.1 absorbances at 350 nm were prepared for visible photoluminescence measurements (**Eu-Cy-TTA** is insoluble in pure water, hence is first dissolved in DMSO and diluted with Milli-Q water.). Measurements were prepared in the unit of absorbance instead of concentration as the absorbances at 350 nm are slightly different for the two complexes. Separate samples were used for (1) UV-vis, emission and excitation scans; (2) luminescence lifetime measurements and (3) quantum yield measurements.

All room temperature solution measurements were done in a quartz cuvette (Starna) of 1 cm path length. UV-vis spectra were recorded with an HP UV-8453 spectrophotometer. Room temperature photoluminescence measurements data obtained with Edinburgh Instruments FLSP920 spectrophotometer equipped with a Xe900 continuous xenon lamp (450 W), xenon flashlamp (60 W) and a Hamamatsu R928P thermoelectrically cooled at –20 °C. Low temperature (77 K) measurements were measured on FLSP920 using an EPR dewar from Edinburgh Instruments. Samples were dissolved in ethanol–methanol mixture (v/v = 4 : 1), inserted into an EPR quartz sample rod and cooled with liquid nitrogen. Emission spectra were recorded at 30 min intervals until the intensity and emission profiles remained constant (∼2 hours) and the spectra were taken as final. Visible emission spectra obtained were corrected for spectral responses.

Luminescence lifetimes of visible emissions were measured with FLSP290 and fitted with Origin. Luminescence quantum yields were measured relative to quinine sulfate in 0.1 M sulfuric acid (*λ*_ex_ = 350 nm, *Φ* = 0.577). All photophysical measurements were averages of triplicate.

The intrinsic quantum yield of the complex was also calculated using the below equations to gain more insight into the sensitization processes:^48^

1 *Φ*LnL = *Φ*LnLn*η*_sens_

2
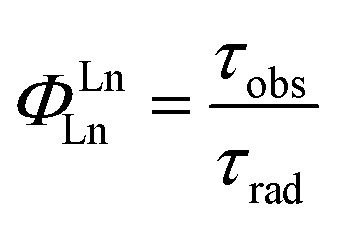

3
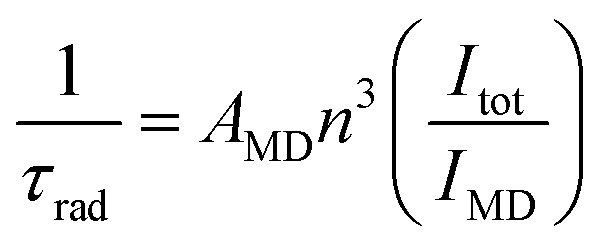

4
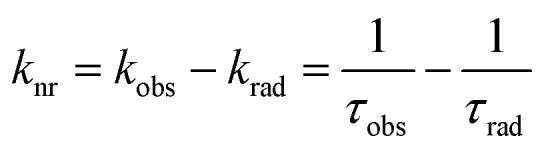



The overall quantum yield (*Φ*LnL) is the product of intrinsic quantum yield (*Φ*LnLn) and sensitization efficiency (*η*_sens_). The reciprocal of the radiative lifetime (1/*τ*_rad_) could be calculated by eqn (3), where AMD denotes the spontaneous emission probability of the magnetic dipole transition (^5^D_0_ → ^7^F_0_ for Eu(iii)) which is a constant equal to 14.65 s^–1^, *n* is the refractive index of the medium and *I*_tot_ and *I*_MD_ are the integrated intensities of the total ^5^D_0_ → ^7^F_J_ transitions and the magnetic dipole transition respectively. The rate of non-radiative decay could be determined by eqn (4).

### Determination of two-photon absorption cross section

For two-photon experiments, the 700 nm pump source was from an optical parametric amplifier (TOPAS-C) of a femtosecond mode-locked Ti:Sapphire laser system (Coherent Micra and Legend-Elite output beam ∼100 fs duration and 100 Hz repetition rate). The laser was focused to spot size ∼100 μm *via* an *f* = 30 cm lens onto the sample. The emitting light was collected with a right angle configuration into a 0.3 m spectrograph and detected by a liquid nitrogen-cooled CCD detector. A power meter was used to monitor the stability of the pump source and its intensity was controlled by using a variable ND filter. For two photon absorption cross-section measurements, the theoretical framework and experimental protocol for the two-photon cross-section measurement have been outlined by Webb and Xu.^49^ In this approach, the two-photon excitation ratios of the reference and sample systems are given by:5
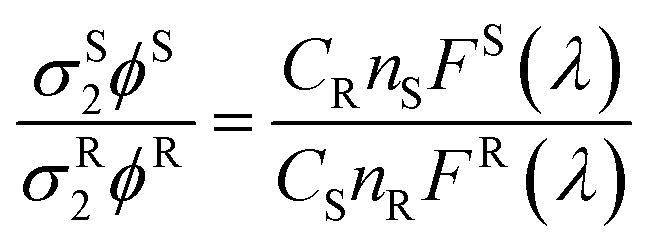
where *φ* is the quantum yield, *C* is the concentration, *n* the refractive index, and *F*(*λ*) is the integrated photoluminescent spectrum. In our measurements, we have ensured that the excitation flux and the excitation wavelengths are the same for both the sample and the reference. The two-photon absorption cross-sections *σ*_2_ of compounds were determined using fluorescein as a reference.^24^ Note the *σ*_2_ values are underestimation of the actual values because only the Eu Δ = *J* bands of 1, 2, 3 were used in the calculations due to the interference of the laser excitation at 700 nm which is in the Δ*J* = 4 band of the Eu transition.

### Cell imaging studies

For single-photon microscopy, images were obtained by a Carl Zeiss AxioObserver Z1 fluorescent microscope using a UV light source. For multi-photon microscopy, images were collected by a Leica TCS SP8 spectral confocal microscope equipped with a Ti:Sapphire laser. Living HeLa cells were used.

### Synthesis

#### Compound **1**

Cyclen (1 g, 5.8 mmol, 1.0 equiv.) was dissolved in acetonitrile (10 mL) and benzene (10 mL), then tosylaziridine (5.5 g, 27.9 mmol, 4.8 equiv.) was added and the mixture was reacted at 60 °C for 3 days. After filtration and washing with acetonitrile, the tosylated intermediate was obtained as a white solid and used for the next step directly without any further purification (4.0 g, 4.2 mmol, yield: 71%). ^1^H NMR (400 MHz, DMSO) *δ* 7.61 (t, *J* = 10.7 Hz, 8H), 7.34 (d, *J* = 7.6 Hz, 8H), 2.72 (t, *J* = 6.5 Hz, 8H), 2.33 (d, *J* = 7.6 Hz, 12H), 2.32–2.20 (m, 24H). ^13^C NMR (100 MHz, DMSO) *δ* 143.00, 138.14, 130.05, 128.79, 54.12, 52.55, 40.80, 21.40. *m*/*z* (ESI-MS^+^) 961.3864 ([M + H]^+^ calculated: 961.3809). Acetic acid (7 mL) and hydrobromic acid (5 mL) were added to dissolve the intermediate (1.0 g, 1.0 mmol, 1.0 equiv.) and the mixture was reacted at 100 °C for 3 days. After cooling down to room temperature, the reaction mixture was filtered and washed with acetic acid and the filter cake was dried at 55 °C in oven to obtain a white solid (300 mg, 0.3 mmol, yield 29%).

#### Compound **3**

Compound **1** (114 mg, 0.11 mmol, 1.0 equiv.) and **2** (242 mg, 0.92 mmol, 8.0 equiv., synthesized according to literature^10^) were dissolved in dry THF (10 mL) and NMM (*N*-methylmorpholine) (184 mg, 1.8 mmol, 16.0 equiv.) was added at room temperature. HATU (1-[Bis(dimethylamino)methylene]-1*H*-1,2,3-triazolo[4,5-*b*]pyridinium 3-oxid hexafluorophosphate) (350 mg, 0.92 mmol, 8.4 equiv.) was added at 4 °C and the reaction mixture was allowed to react at room temperature overnight before quenching with water (50 mL). The organic solvent was evaporated and the aqueous layer was discarded. The residual oil adhered to the wall of flask was washed with water (30 mL) again and purified by preparative HPLC to obtain the product (80 mg, 0.064 mmol, yield: 55.5%). ^1^H NMR (400 MHz, CD_3_OD) *δ* 7.88–7.04 (m, 24H), 6.72 (d, *J* = 9.0 Hz, 4H), 6.41 (d, *J* = 6.5 Hz, 1H), 5.27 (s, 8H), 3.54 (s, 8H), 3.21–2.78 (m, 24H). ^13^C NMR (100 MHz, CD_3_OD) *δ* 161.79, 159.16, 142.56, 139.16, 133.63, 129.93, 129.30, 128.51, 123.15, 106.17, 79.00, 52.10, 48.6, 34.30. *m*/*z* (ESI-MS^+^) 1253 ([M + H]^+^ calculated: 1253).

#### Compound **4**

Compound **3** (80 mg, 0.064 mmol, 1.0 equiv.) was dissolved in acetic acid (3 mL) and hydrochloric acid (3 mL) and the reaction mixture was stirred at room temperature for 3 days. The solution was then concentrated and dissolved in methanol (0.5 mL). The product was precipitated by slow addition of diethyl ether and collected by centrifugation in the form of an HCl salt (65 mg, 0.063 mmol, yield: 98%). ^1^H NMR (400 MHz, D_2_O) *δ* 7.44 (t, *J* = 7.9 Hz, 4H), 6.69 (d, *J* = 8.9 Hz, 4H), 6.61 (d, *J* = 6.7 Hz, 4H), 3.63 (s, 8H), 3.30 (s, 16H), 3.20 (s, 8H). ^13^C NMR (100 MHz, D_2_O) *δ* 162.79, 159.85, 139.56, 139.03, 121.14, 108.86, 52.14, 49.00, 35.05. *m*/*z* (ESI-MS^+^) 893.3911 ([M + H]^+^ calculated: 893.3906).

#### **Ln-Cy-HOPO**

Compound **4** (15 mg, 0.014 mmol, 1.0 equiv.) was dissolved in methanol (1 mL) and a methanol (1 mL) solution of LnCl_3_·6H_2_O (0.015 mmol, 1.05 equiv.) was added. The pH of the solution was adjusted to 7.5 by pyridine. The solution was stirred at 55 °C for 12 hours. The product was precipitated by slow addition of diethyl ether (5 mL), and the solids were collected by centrifugation, washed twice with diethyl ether (5 mL x 2) and dried as white solids (∼80% yields). **Eu-Cy-HOPO** (12.5 mg, yield: 83%): *m*/*z* (ESI-MS^+^) 1043.2893 ([M + 2H]^+^ calculated: 1043.2883); calcd Eu content 14.6% (C_40_H_49_EuN_12_O_12_), found 13.9%. **Sm-Cy-HOPO** (12.9 mg, yield: 86%): *m*/*z* (ESI-MS^+^) 1042.2863 ([M+2H]^+^ calculated: 1042.2865); calcd Sm content 14.5% (C_40_H_49_SmN_12_O_12_), found 13.3%. **Gd-Cy-HOPO** (11.8 mg, yield: 78%): *m*/*z* (ESI-MS^+^) 1048.2914 ([M + 2H]^+^ calculated: 1048.2916); calcd Gd content 15.0% (C_40_H_49_GdN_12_O_12_), found 14.3%.

#### Compound **5**

Cyclen (202 mg, 1.16 mmol 1.0 equiv.) was dissolved in dried acetonitrile (4 mL). Potassium carbonate (660 mg, 4.78 mmol, 4.1 equiv.) and 2-acetyl-4-chloromethyl-thiophene (806 mg, 4.62 mmol, 4.0 equiv.) were added into the solution and the reaction mixture was stirred at room temperature for 24 hours, before increasing the temperature to 40 °C and allowed to react for another 2 days. The reaction mixture was then cooled, filtered and dried under vacuum. The product was purified by column chromatography on silica gel (CHCl_3_ : EtOH, 100 : 1–10 : 1) to obtain compound **5** as a light yellow oil (550 mg, 0.76 mmol) with a 65% yield. ^1^H NMR (400 MHz, CDCl_3_) *δ* 7.52 (d, *J* = 16.5 Hz, 8H), 3.42 (s, 8H), 2.63 (s, 16H), 2.42 (s, 12H). ^13^C NMR (100 MHz, CDCl_3_) *δ* 190.65, 144.22, 141.91, 133.45, 130.96, 54.63, 52.93, 26.80. m/*z* (ESI-MS^+^) 725.2363 ([M + H]^+^ calculated: 725.2324).

#### Compound **6**

Compound **5** (51.6 mg, 0.07 mmol, 1.0 equiv.) was dissolved in dried THF (4 mL) and cooled to –78 °C. Potassium bis(trimethylsilyl)amide (KHMDS) (1.0 M in THF, 0.43 mL, 0.42 mmol, 6.0 equiv.) was dropped slowly into the reaction mixture and stirred for 20 minutes. Ethyl trifluoroacetate (60.7 mg, 0.43 mmol, 6.1 equiv.) was then added and the reaction was continually stirred at –78 °C for 2 more hours before stirring overnight at room temperature. Petroleum ether (5 mL) was added to the solution and stirred for 20 minutes to give precipitates, which were collected by centrifugation and further washed with dichloromethane twice (13 mL × 2). The product was obtained as an orange solid after drying under vacuum (60 mg, 0.05 mmol, yield: 67%). ^1^H NMR (400 MHz, DMSO) *δ* 7.85–7.35 (m, 8H), 6.09–5.73 (m, 4H), 3.93–3.3 (s, 4H), 3.58–3.42 (m, 4H), 2.65–2.61 (m, 8H), 2.48–2.42 (m, 8H). ^19^F NMR (376 MHz, DMSO) *δ* –73.49 (6F), –7.72 (6F). m/*z* (ESI-MS^+^) 1109.1645 ([M – 4K + 5H]^+^ calculated: 1109.1620).

#### **Ln-Cy-TTA**

Compound **6** (12 mg, 0.0095 mmol, 1.0 equiv.) was dissolved in methanol (1 mL) and a methanol solution (1 mL) of LnCl_3_·6H_2_O (0.01 mmol, 1.1 equiv.) was added. The reaction mixture was stirred for 16 hours at 50 °C. The product was obtained by precipitating with water (6 mL), centrifuged and washed with water twice more (6 mL × 2). A dried yellow/orange solid was obtained as the final complex (∼80% yields) with a counter ion of potassium. **Eu-Cy-TTA** (10 mg, yield: 81%): *m*/*z* (ESI-MS^+^) 1259.0579 ([M – K + 2H]^+^ calculated: 1259.0585); calcd Eu content 11.7% (C_44_H_36_F_12_EuKN_4_O_8_S_4_), found 12.1%. **Gd-Cy-TTA** (10 mg, yield: 81%): *m*/*z* (ESI-MS^+^) 1264.0605 ([M – K + 2H]^+^ calculated: 1264.0619); calcd Gd content 12.1% ((C_44_H_36_F_12_GdKN_4_O_8_S_4_), found 12.5%.

## Conflicts of interest

There are no conflicts to declare.

## Supplementary Material

Supplementary information
