# Supplementary material for: Breaking the 1,2-HOPO barrier with a cyclen backbone for more efficient sensitization of Eu(iii) luminescence and unprecedented two-photon excitation properties
Source: Chem Sci. 2019 Mar 28;10(17):4550–9. doi: 10.1039/c9sc00244h (PMC6498141; doi:10.1039/c9sc00244h)
Supplement: Supplementary file 1 [file SC-010-C9SC00244H-s001.pdf]

Electronic Supplementary Material (ESI) for Chemical Science.
This journal is © The Royal Society of Chemistry 2019

Supporting Information

Breaking the 1,2-HOPO barrier with a cyclen backbone for more efficient sensitization of Eu(III) luminescence and unprecedented two-photon excitation properties

Lixiong Dai[abc†], Wai-Sum Lo[ab†], Yanjuan Gu[b], Qingwu Xiong[b], Ka-Leung Wong[c], Wai-Ming Kwok[b], Wing-Tak Wong*[ab], Ga-Lai Law*[ab]

[a]*The Hong Kong Polytechnic University Shenzhen Research Institute, Shenzhen, PR China.*

[b]*Department of Applied Biology and Chemical Technology, The Hong Kong Polytechnic University, Hung Hom, Kowloon, Hong Kong SAR, PR China.*

[c]*Department of Chemistry, Hong Kong Baptist University, Kowloon Tong, Hong Kong SAR, PR China.*

E-mail: w.t.wong@polyu.edu.hk; ga-lai.law@polyu.edu.hk

Contents

Figure S1. Luminescence decay curve of $^5D_0 \rightarrow ^7F_2$ transition of **Eu-Cy-HOPO** in water (λ_{ex} = 350 nm, λ_{em} = 614 nm). 1

Figure S2. Luminescence decay curve of $^5D_0 \rightarrow ^7F_2$ transition of **Eu-Cy-HOPO** in water (λ_{ex} = 350 nm, λ_{em} = 614 nm). 1

Figure S3. Luminescence decay curve of $^5D_0 \rightarrow ^7F_2$ transition of **Eu-Cy-TTA** in water (λ_{ex} = 350 nm, λ_{em} = 613 nm). 2

Figure S4. Emission spectra of **Gd-Cy-HOPO** at room temperature (red) and 77K (black) in water/glycerol (1:1) mixture (λ_{ex} = 350 nm). 2

Figure S5. Phosphorescence decay curve of **Gd-Cy-HOPO** in water:glycerol (1:1) mixture (λ_{ex} = 350 nm, λ_{em} = 503 nm). 3

Figure S6. Emission spectra of **Gd-Cy-TTA** at room temperature (red) and 77K (black) in 2-methyltetrahydrofuran (λ_{ex} = 350 nm). 3

Figure S7. Phosphorescence decay curve of **Gd-Cy-TTA** in 2-methyltetrahydrofuran (λ_{ex} = 350 nm, λ_{em} = 510 nm). 4

Figure S8. Phosphorescence decay curve of **Gd-Cy-TTA** in 2-methyltetrahydrofuran (λ_{ex} = 350 nm, λ_{em} = 534 nm). 4

Figure S9. Mass spectrum of ligand **4**. Ion peaks at 447.2003 and 893.3911 (m/z) which correspond to $[M+2H]^{2+}/2$ and $[M+H]^+$ respectively. 5

Figure S10. Mass spectrum of ligand **6**. Ion peaks at 1109.1645, 1131.1353, 1147.1111, 1153.1153, and 1169.0929 (m/z) which correspond to $[M-4K+5H]^+$, $[M-4K+4H+Na]^+$, $[M-3K+4H]^+$, $[M-4K+3H+2Na]^+$, $[M-3K+3H+Na]^+$ respectively. 5

Figure S11. Mass spectrum of **Eu-Cy-HOPO**. Ion peaks at 522.1489 and 1043.2893 (ESI m/z) which correspond to $[M+3H]^{2+}/2$ and $[M+2H]^+$ respectively, ion peak at 1043.2870 (MALDI m/z) which corresponds to $[M+2H]^+$. 6

Figure S12. Mass spectrum of **Sm-Cy-HOPO**. Ion peaks at 521.6481 and 1042.2863 (ESI m/z) which correspond to $[M+3H]^{2+}/2$ and $[M+2H]^+$ respectively, ion peak at 1042.2879 (MALDI m/z) which corresponds to $[M+2H]^+$. 6

Figure S13. Mass spectrum of **Gd-Cy-HOPO**. Ion peak 1048.2914 (ESI m/z) which corresponds to $[M+2H]^+$, ion peak at 1048.2901 (MALDI m/z) which corresponds to $[M+2H]^+$. 7

Figure S14. Mass spectrum of **Eu-Cy-TTA**. Ion peaks 1259.0579 and 1281.0399 (m/z) which correspond to $[M-K+2H]^+$ and $[M-K+H+Na]^+$ respectively. 7

Figure S15. Mass spectrum of **Gd-Cy-TTA**. Ion peak 1264.0605 (m/z) which corresponds to $[M-K+2H]^+$. 8

Figure S16. ^1H NMR spectrum of intermediate of compound **1** in $CDCl_3$. 8

Figure S17. ^1H NMR spectrum of intermediate of compound **1** in d^6-DMSO. 9

Figure S18. ^{13}C NMR spectrum of intermediate of compound **1** in d^6-DMSO. 9

Figure S19. ^1H NMR spectrum of compound **1** in D_2O. 10

Figure S20. ^{1}H NMR spectrum of compound **3** in CD_3OD. 10

Figure S21. ^{13}C NMR spectrum of compound **3** in CD_3OD. 11

Figure S22. ^{1}H NMR spectrum of compound **4** in D_2O. 11

Figure S23. ^{1}C NMR spectrum of compound **4** in D_2O. 12

Figure S24. ^{1}H NMR spectrum of complex **Eu-Cy-HOPO** in D_2O. 12

Figure S25. ^{1}H NMR spectrum of compound **5** in $CDCl_3$. 13

Figure S26. ^{13}C NMR spectrum of compound **5** in $CDCl_3$. 13

Figure S27. ^{1}H NMR spectrum of compound **6** in d^6-DMSO. 14

Figure S28. ^{13}C NMR spectrum of compound **6** in d^6-DMSO. 14

Figure S29. ^{19}F NMR spectrum of compound **6** in d^6-DMSO. 15

Figure S30. RP-HPLC trace of complex **Eu-Cy-HOPO** (350 nm) 15

Figure S31. RP-HPLC trace of complex **Sm-Cy-HOPO** (350 nm). 16

Figure S32. RP-HPLC trace of complex **Gd-Cy-HOPO** (350 nm). 16

Figure S33. Comparison of IR spectra of ligand **4** and complexes of **Eu-Cy-HOPO**, **Sm-Cy-HOPO** and **Gd-Cy-HOPO**. 17

Figure S34. Comparison of IR spectra of ligand **6** and complexes of **Eu-Cy-TTA** and **Gd-Cy-TTA**. 17

Figure S35. Dependence of luminescence intensity on incident power of **Eu-Cy-HOPO** in DMSO. 18

Figure S36. Two-photon excitation emission spectra of Fluorescein at pH 12 and **Eu-Cy-HOPO** in DMSO. 18

Figure S37. Dependence of luminescence intensity on incident power of **Eu-Cy-TTA** in DMSO. 19

Figure S38. Two-photon excitation emission spectra of Fluorescein at pH 12 and **Eu-Cy-TTA** in DMSO. 19

Figure S39. Viability of HeLa cells incubated with **Eu-Cy-HOPO** for 24 hours 20

Figure S40. Bright field (left) and fluorescent microscopy image (middle) and overlaid image of **Sm-Cy-HOPO** (40 μM) in HeLa cells after 3 hours of incubation. 20

Figure S41. Optimized structure of **Sm-Cy-HOPO**. View from side (a); above cyclen backbone (b); view from below Sm(III) center (c). 20

Figure S42. Bright field (left) and multi-photon microscopy image (middle) and overlaid image (right) of **Eu-Cy-HOPO** (4 μM) after 3 hours of incubation (λ_{ex} = 760 nm). 21

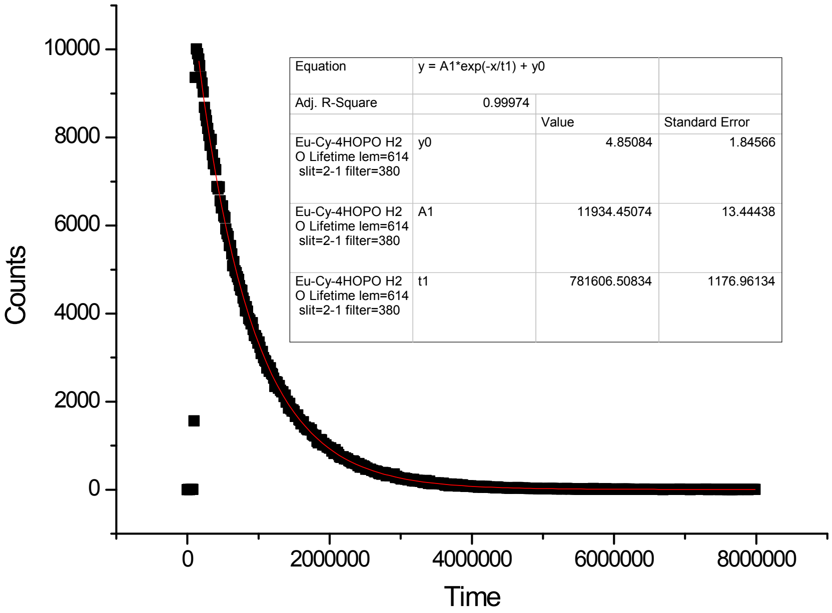

Figure S1. Luminescence decay curve of $^5D_0 \rightarrow {}^7F_2$ transition of **Eu-Cy-HOPO** in water (λ_{ex} = 350 nm, λ_{em} = 614 nm).

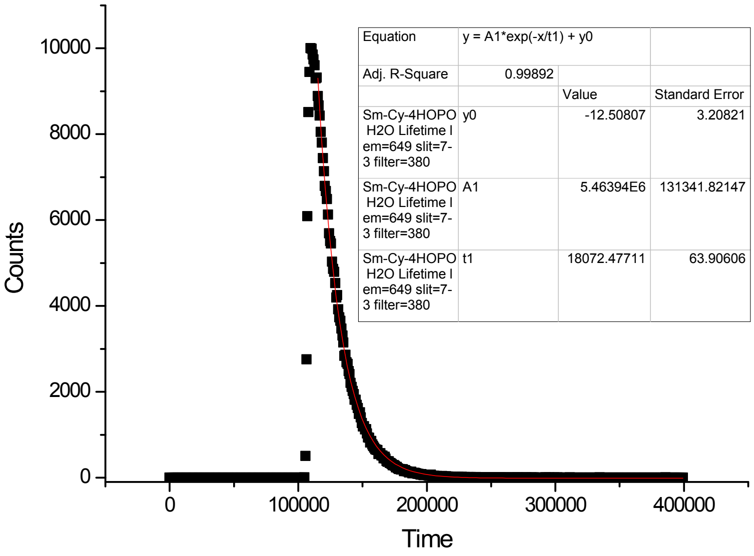

Figure S2. Luminescence decay curve of $^5D_0 \rightarrow {}^7F_2$ transition of **Eu-Cy-HOPO** in water (λ_{ex} = 350 nm, λ_{em} = 614 nm).

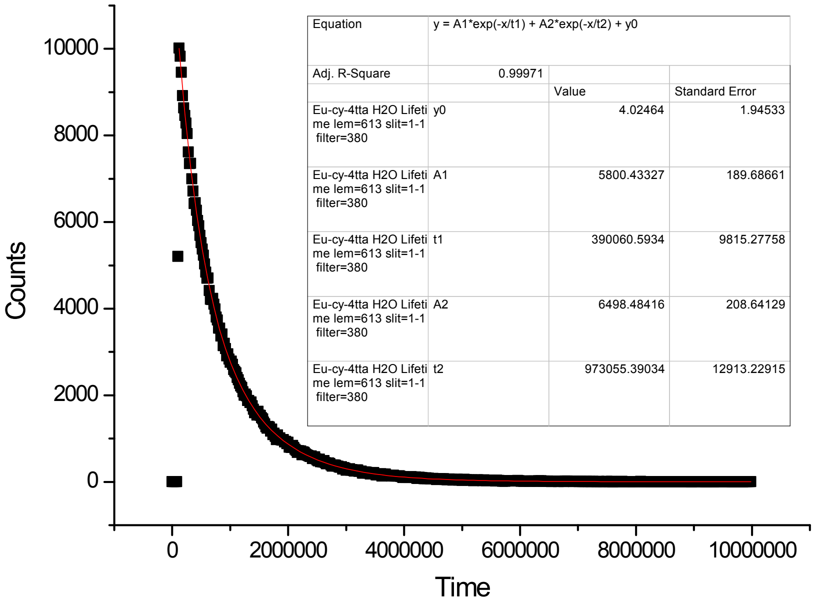

Figure S3. Luminescence decay curve of $^5D_0 \rightarrow {}^7F_2$ transition of **Eu-Cy-TTA** in water (λ_{ex} = 350 nm, λ_{em} = 613 nm).

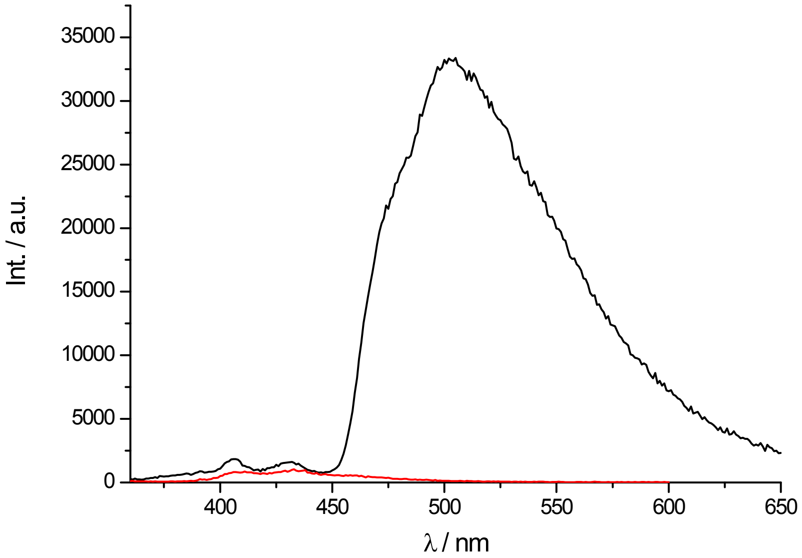

Figure S4. Emission spectra of **Gd-Cy-HOPO** at room temperature (red) and 77K (black) in water/glycerol (1:1) mixture (λ_{ex} = 350 nm).

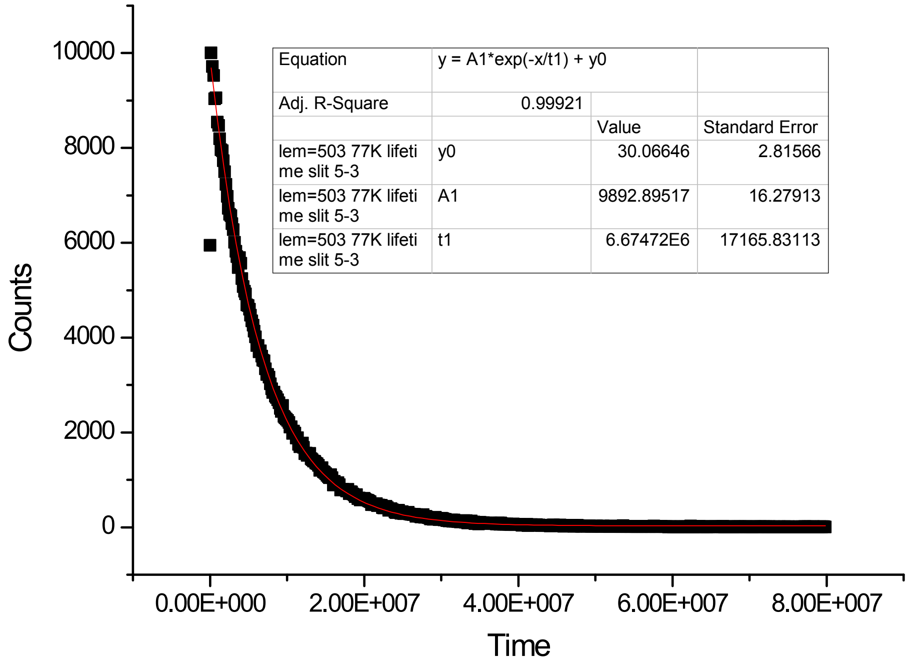

Figure S5. Phosphorescence decay curve of **Gd-Cy-HOPO** in water:glycerol (1:1) mixture (λ_{ex} = 350 nm, λ_{em} = 503 nm).

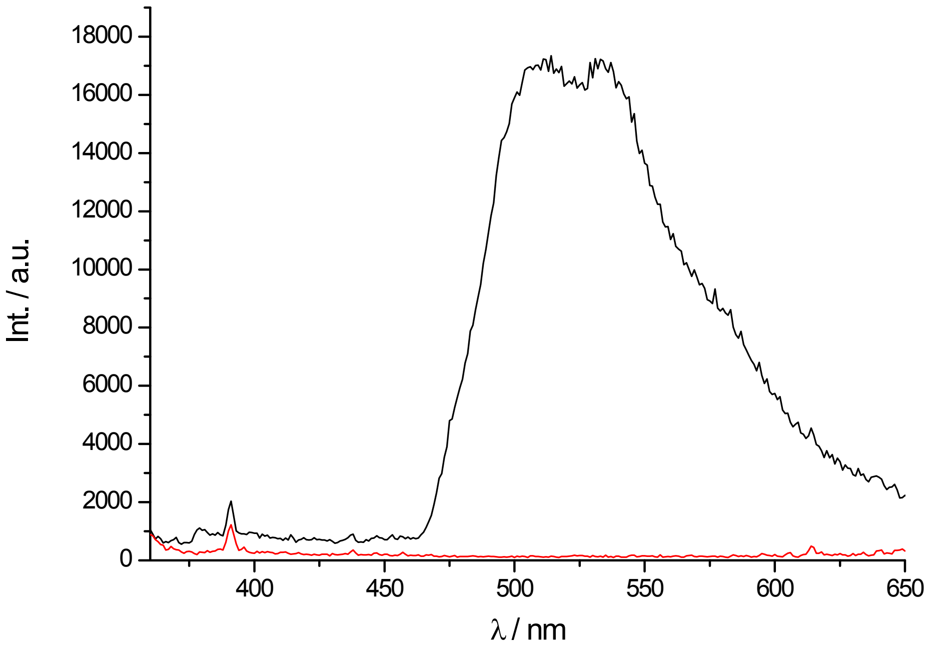

Figure S6. Emission spectra of **Gd-Cy-TTA** at room temperature (red) and 77K (black) in 2-methyltetrahydrofuran (λ_{ex} = 350 nm).

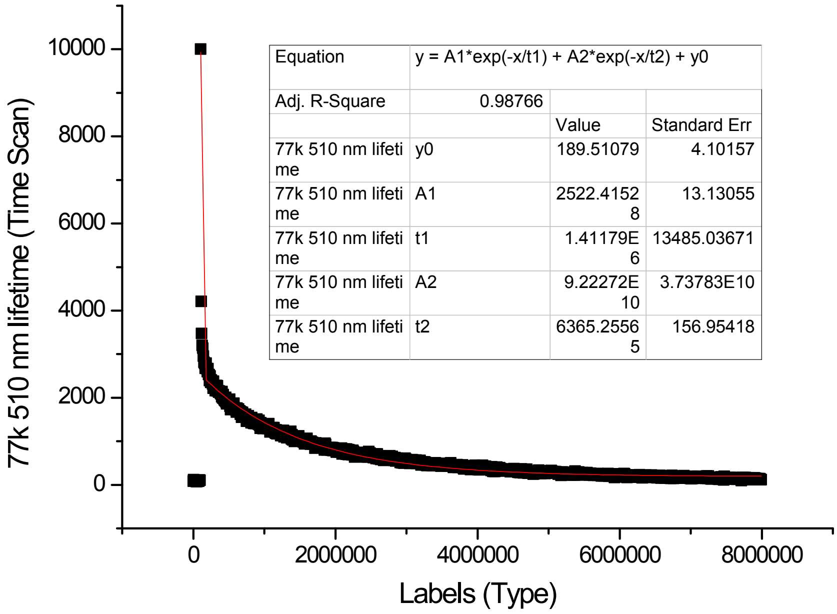

Figure S7. Phosphorescence decay curve of **Gd-Cy-TTA** in 2-methyltetrahydrofuran (λ_{ex} = 350 nm, λ_{em} = 510 nm).

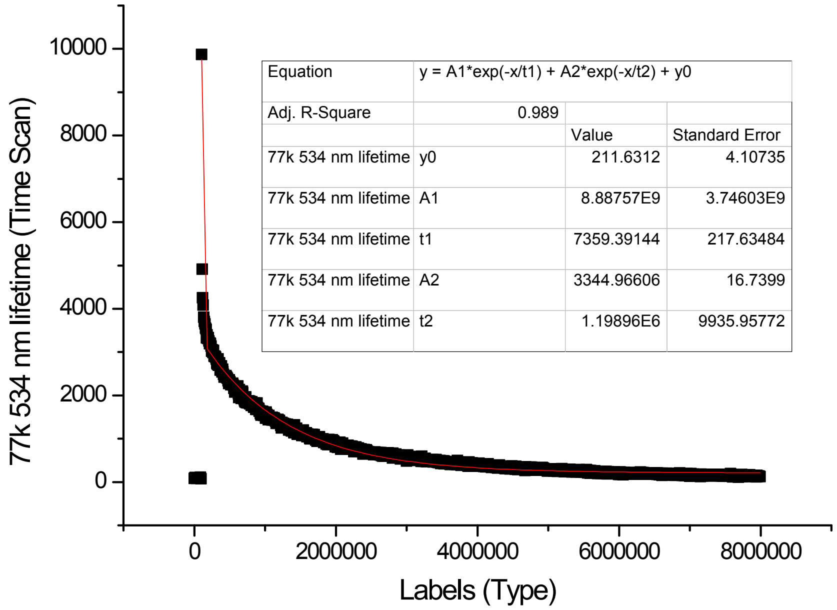

Figure S8. Phosphorescence decay curve of **Gd-Cy-TTA** in 2-methyltetrahydrofuran (λ_{ex} = 350 nm, λ_{em} = 534 nm).

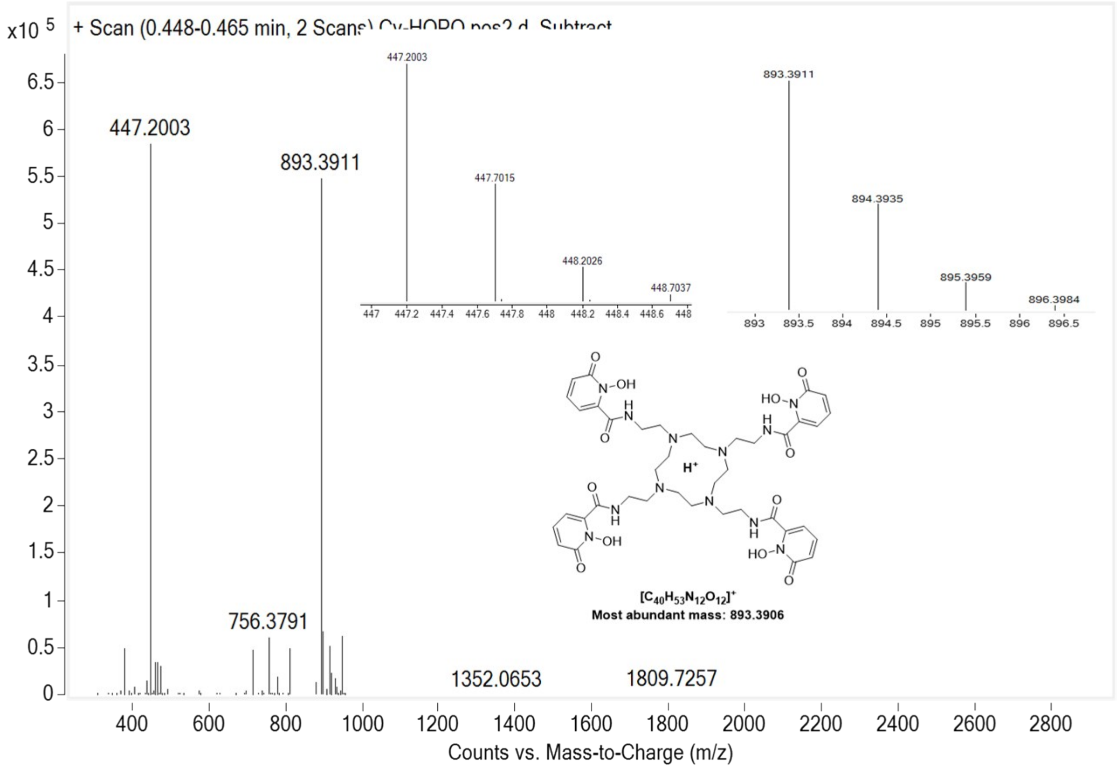

Figure S9. Mass spectrum of ligand **4**. Ion peaks at 447.2003 and 893.3911 (m/z) which correspond to $[M+2H]^{2+}/2$ and $[M+H]^{+}$ respectively.

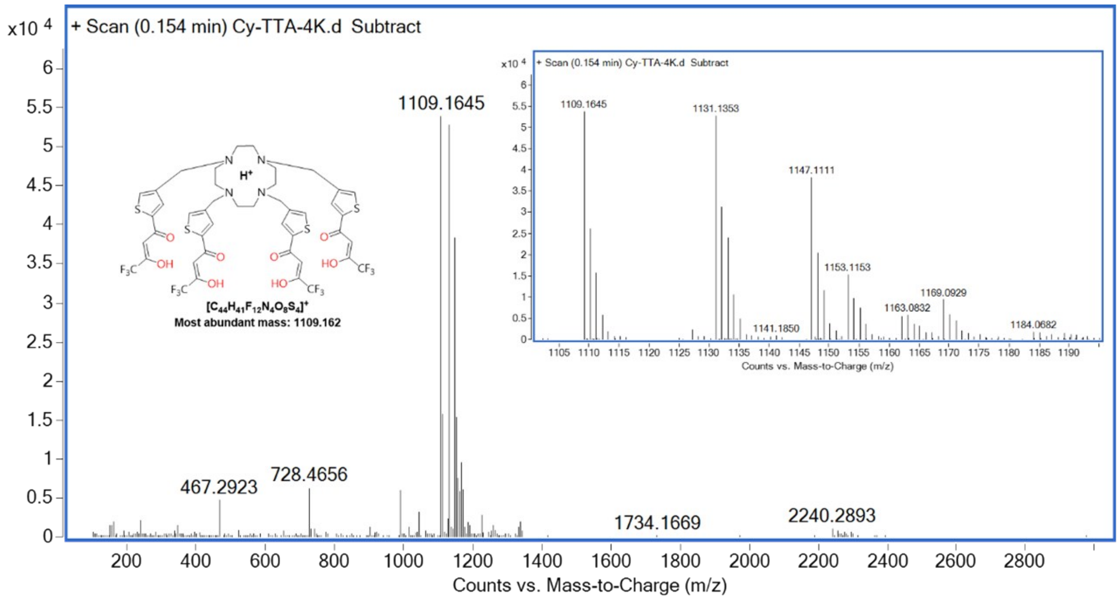

Figure S10. Mass spectrum of ligand **6**. Ion peaks at 1109.1645, 1131.1353, 1147.1111, 1153.1153, and 1169.0929 (m/z) which correspond to $[M-4K+5H]^{+}$, $[M-4K+4H+Na]^{+}$, $[M-3K+4H]^{+}$, $[M-4K+3H+2Na]^{+}$, $[M-3K+3H+Na]^{+}$ respectively.

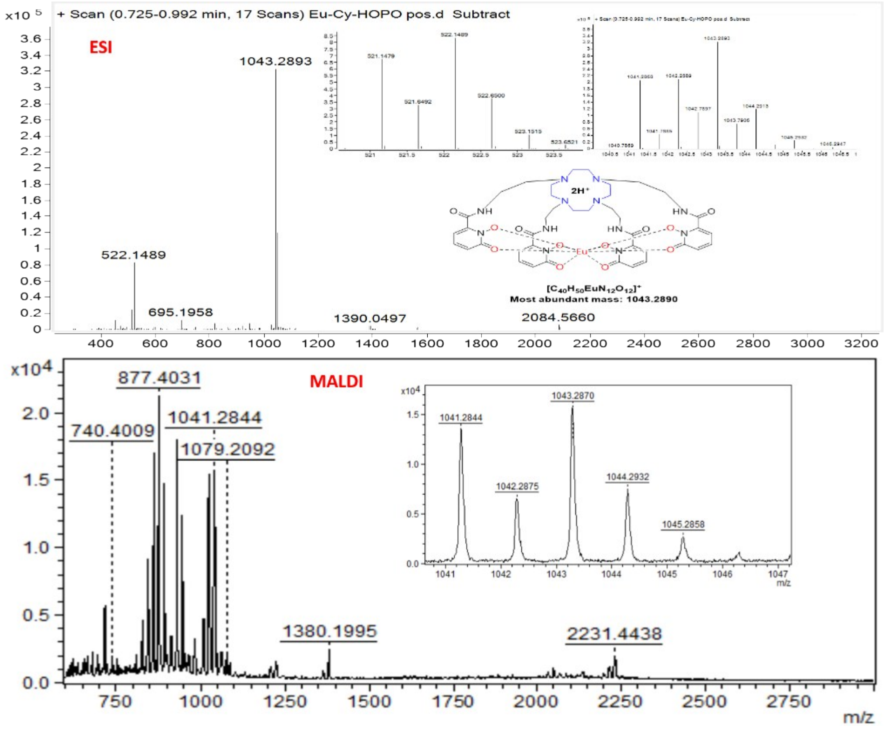

Figure S11. Mass spectrum of **Eu-Cy-HOPO**. Ion peaks at 522.1489 and 1043.2893 (ESI m/z) which correspond to $[M+3H]^{2+}/2$ and $[M+2H]^+$ respectively, ion peak at 1043.2870 (MALDI m/z) which corresponds to $[M+2H]^+$.

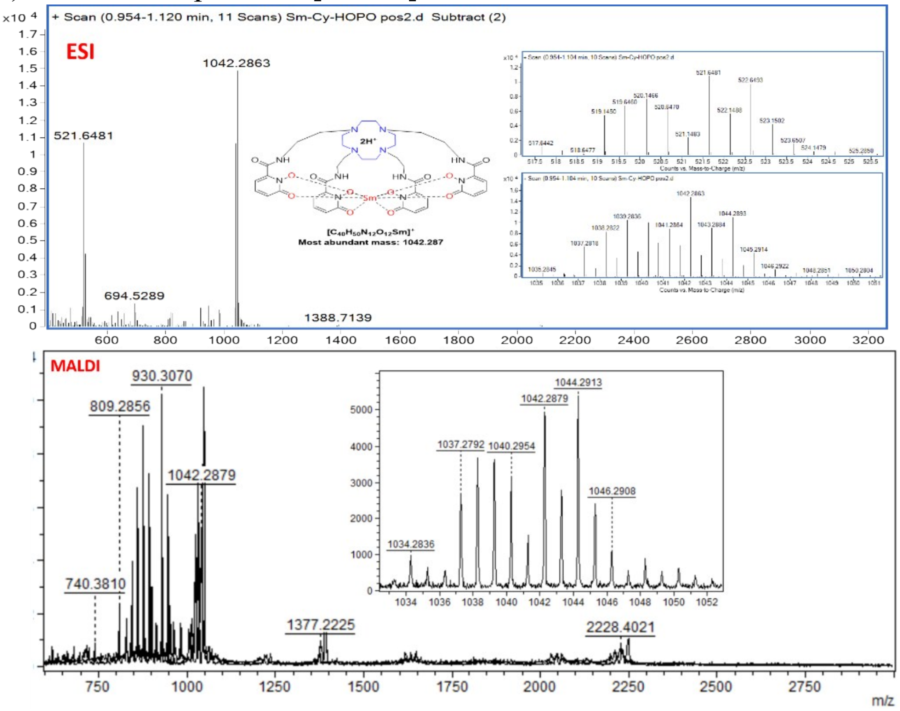

Figure S12. Mass spectrum of **Sm-Cy-HOPO**. Ion peaks at 521.6481 and 1042.2863 (ESI m/z) which correspond to $[M+3H]^{2+}/2$ and $[M+2H]^+$ respectively, ion peak at 1042.2879 (MALDI m/z) which corresponds to $[M+2H]^+$.

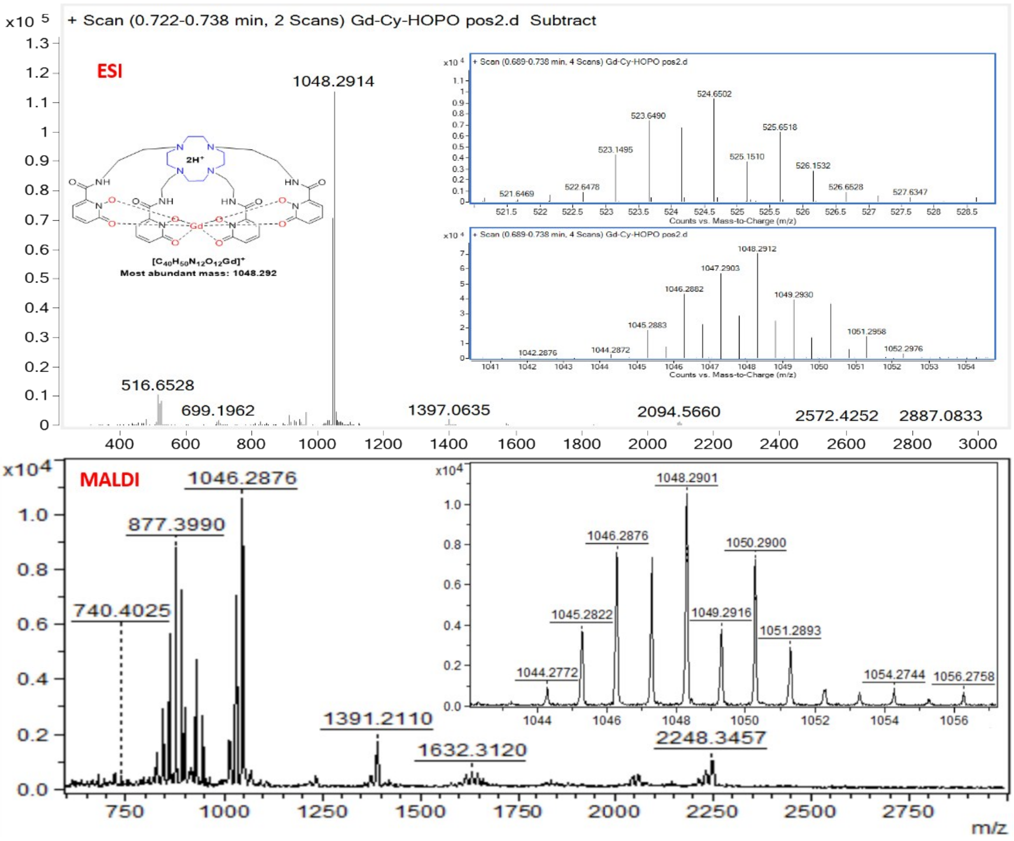

Figure S13. Mass spectrum of **Gd-Cy-HOPO**. Ion peak 1048.2914 (ESI m/z) which corresponds to $[M+2H]^+$, ion peak at 1048.2901 (MALDI m/z) which corresponds to $[M+2H]^+$.

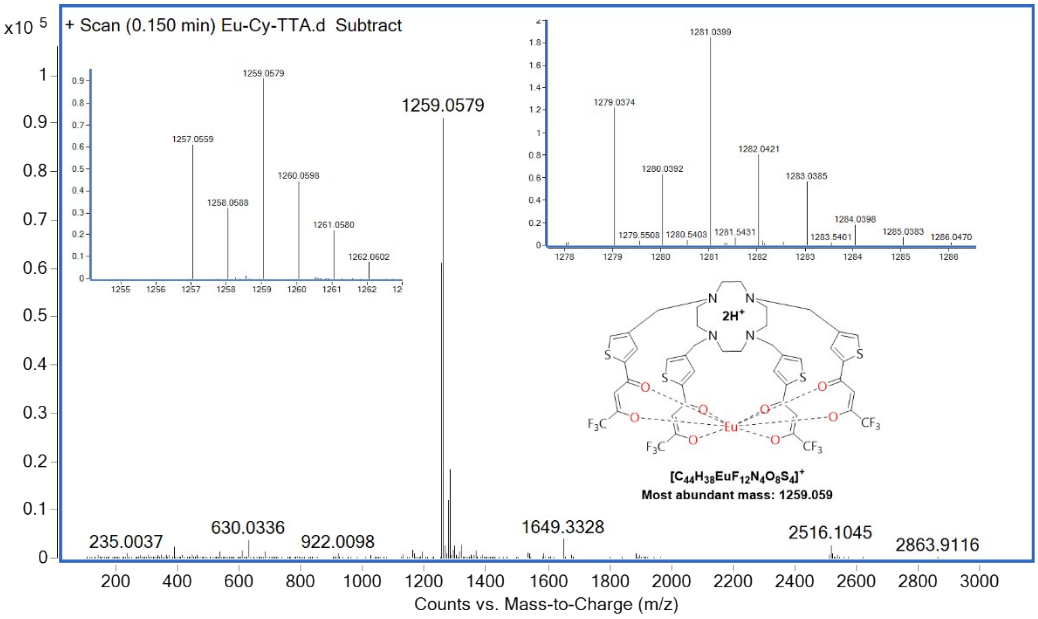

Figure S14. Mass spectrum of **Eu-Cy-TTA**. Ion peaks 1259.0579 and 1281.0399 (m/z) which correspond to $[M-K+2H]^+$ and $[M-K+H+Na]^+$ respectively.

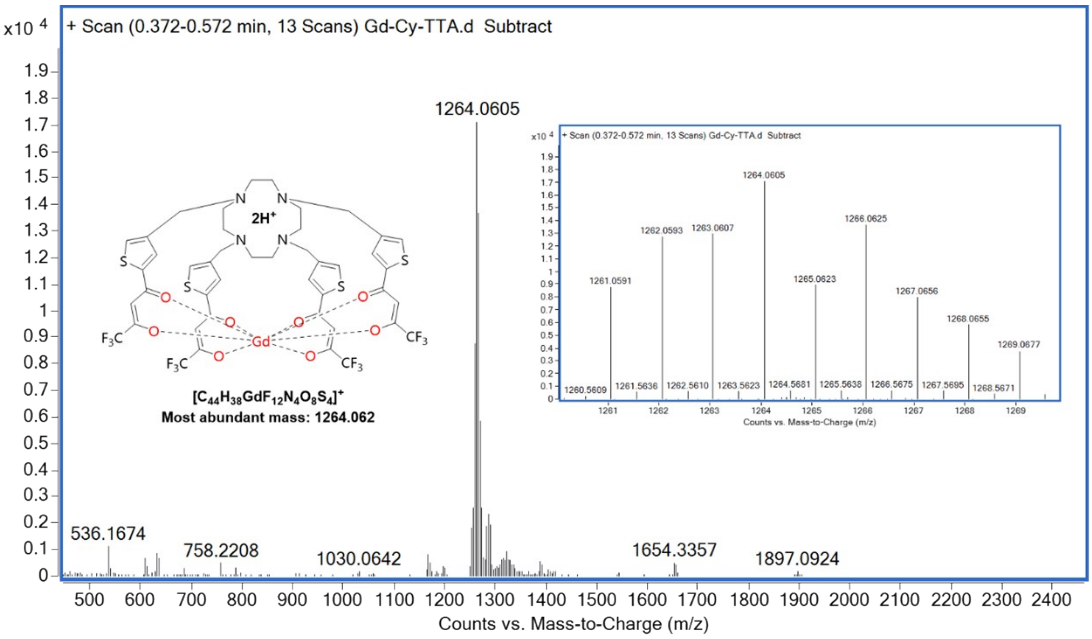

Figure S15. Mass spectrum of **Gd-Cy-TTA**. Ion peak 1264.0605 (m/z) which corresponds to $[M\text{-}K+2H]^+$.

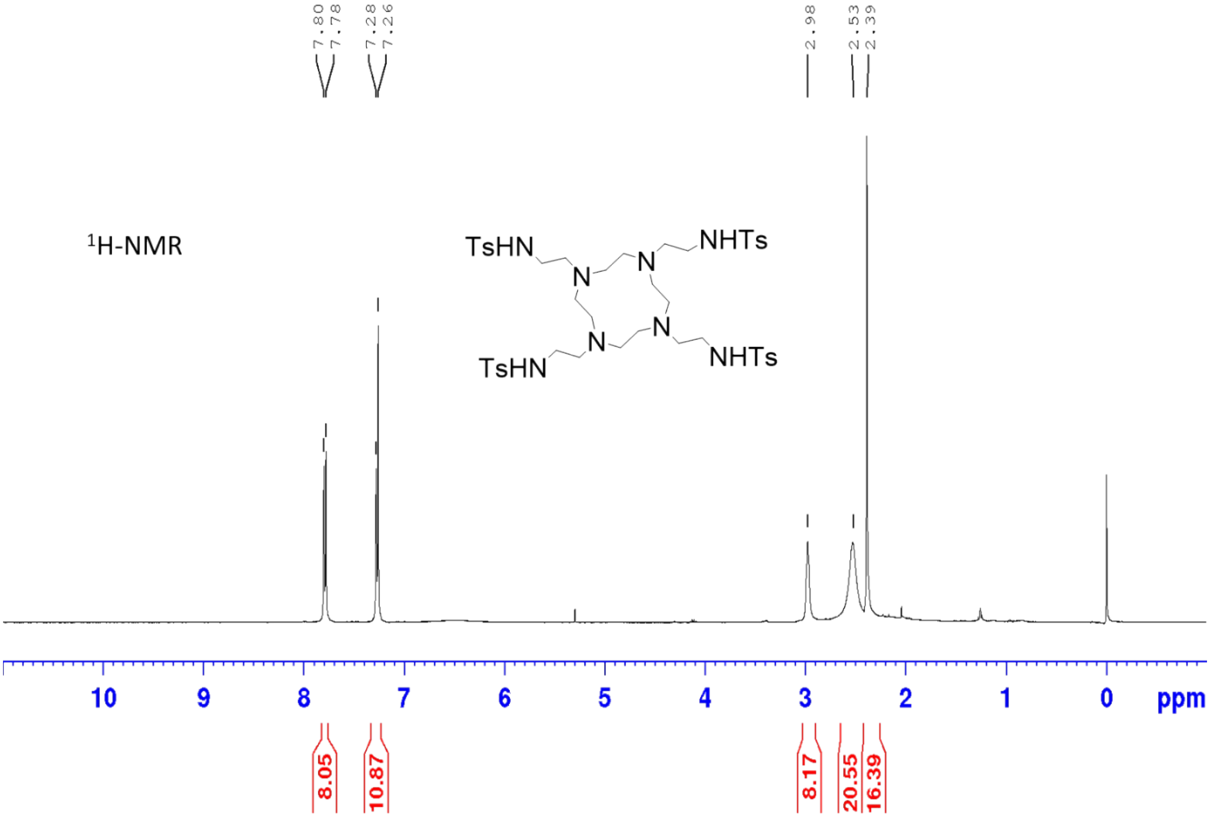

Figure S16. ^1H NMR spectrum of intermediate of compound **1** in $CDCl_3$.

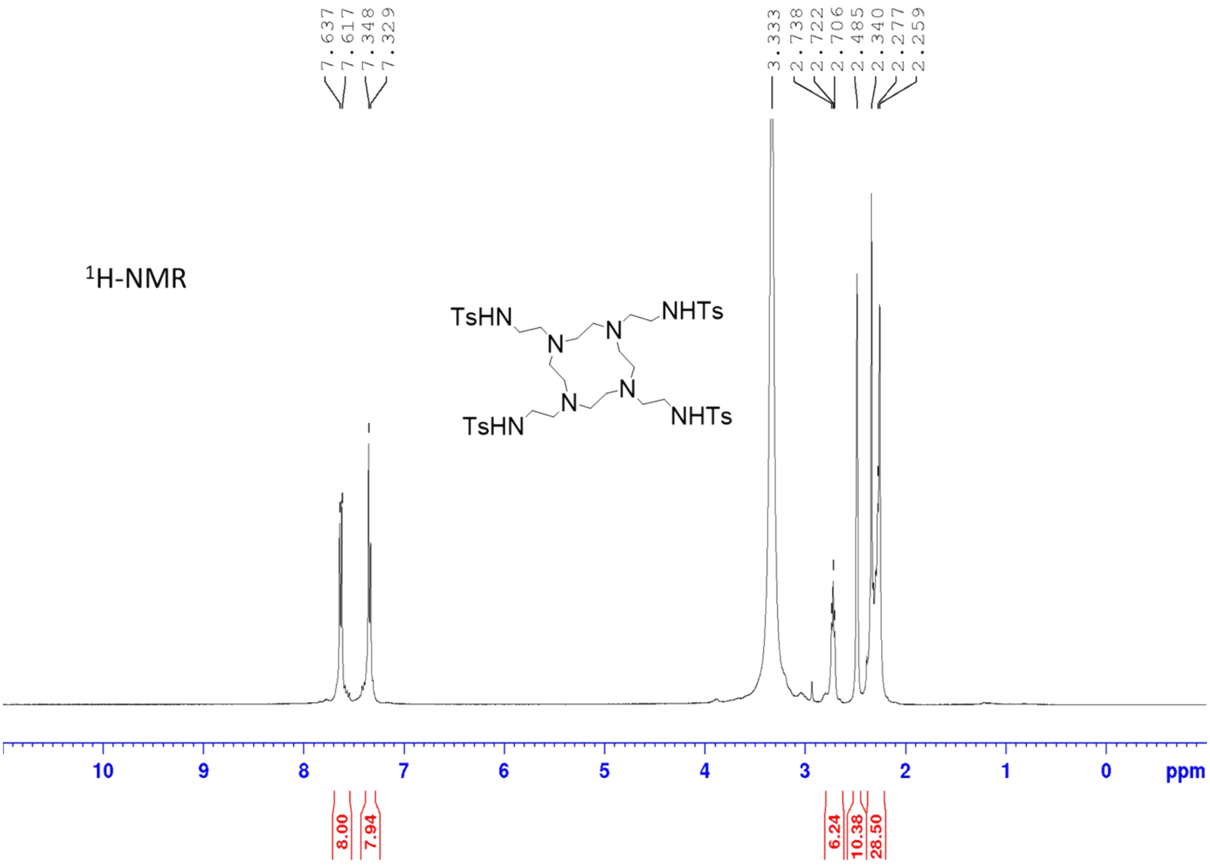

Figure S17. ^{1}H NMR spectrum of intermediate of compound **1** in d^{6}-DMSO.

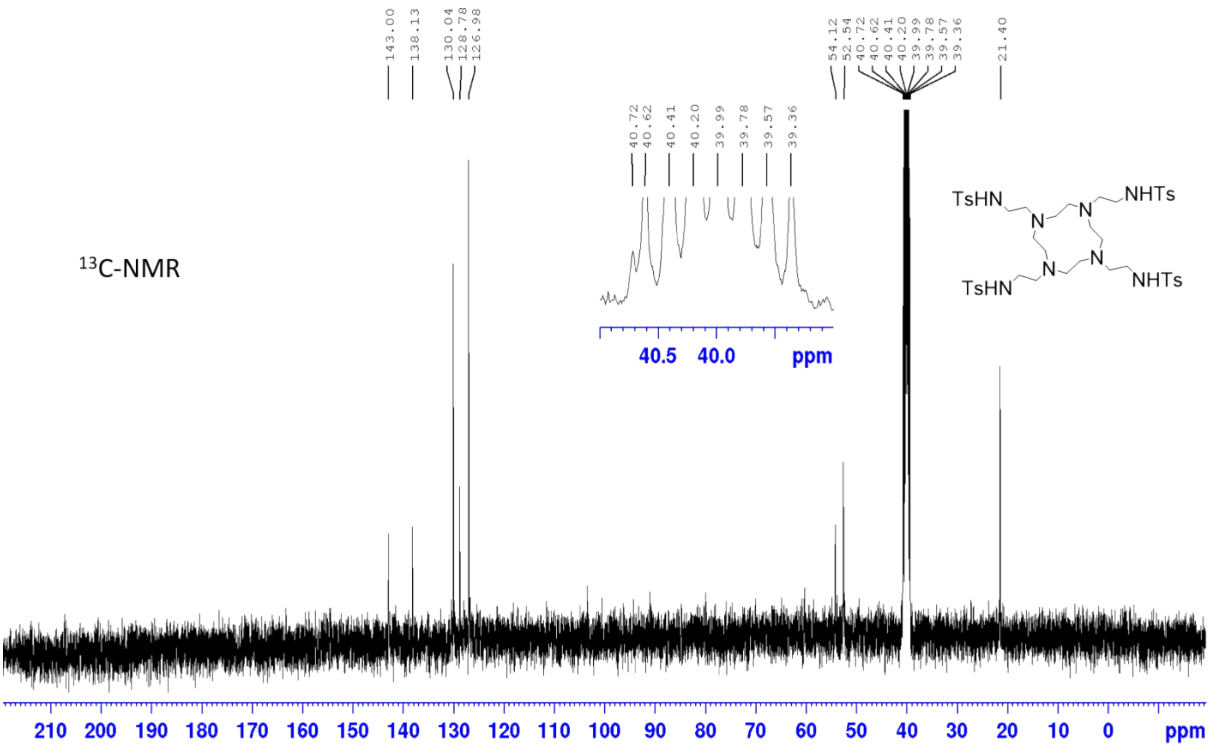

Figure S18. ^{13}C NMR spectrum of intermediate of compound **1** in d^{6}-DMSO.

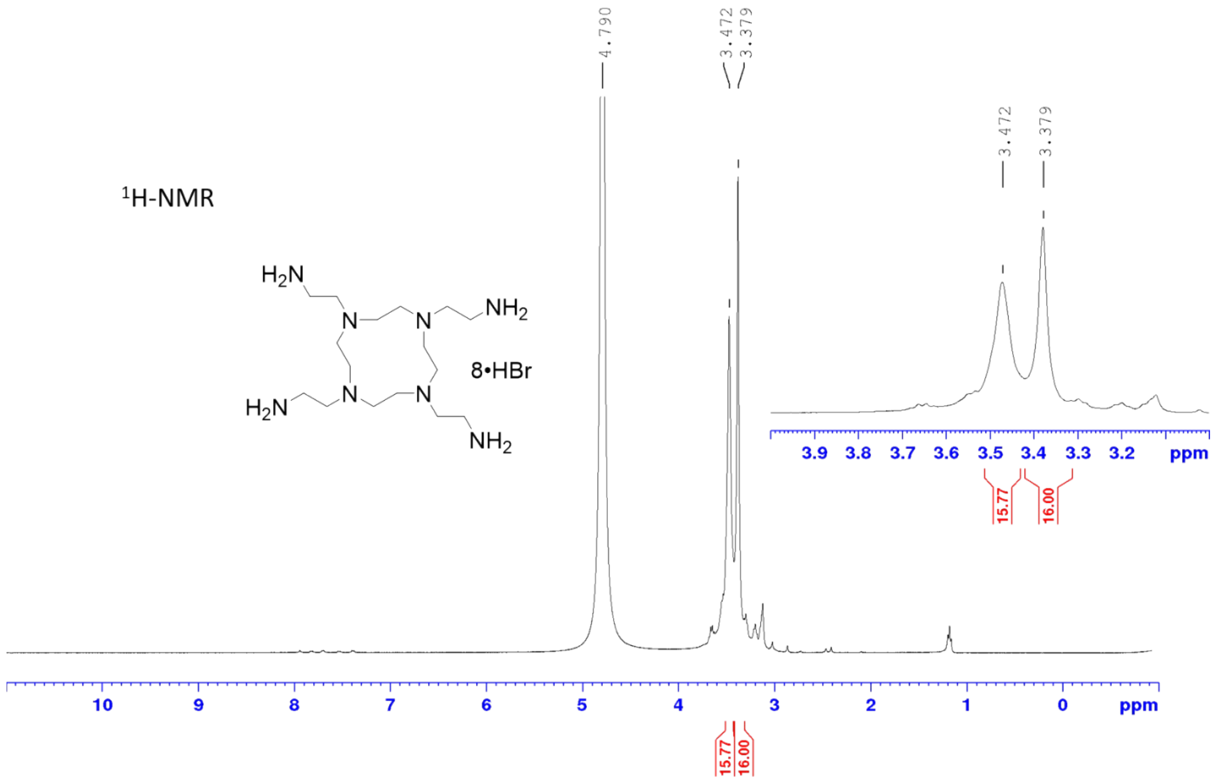

Figure S19. ^{1}H NMR spectrum of compound **1** in D_2O.

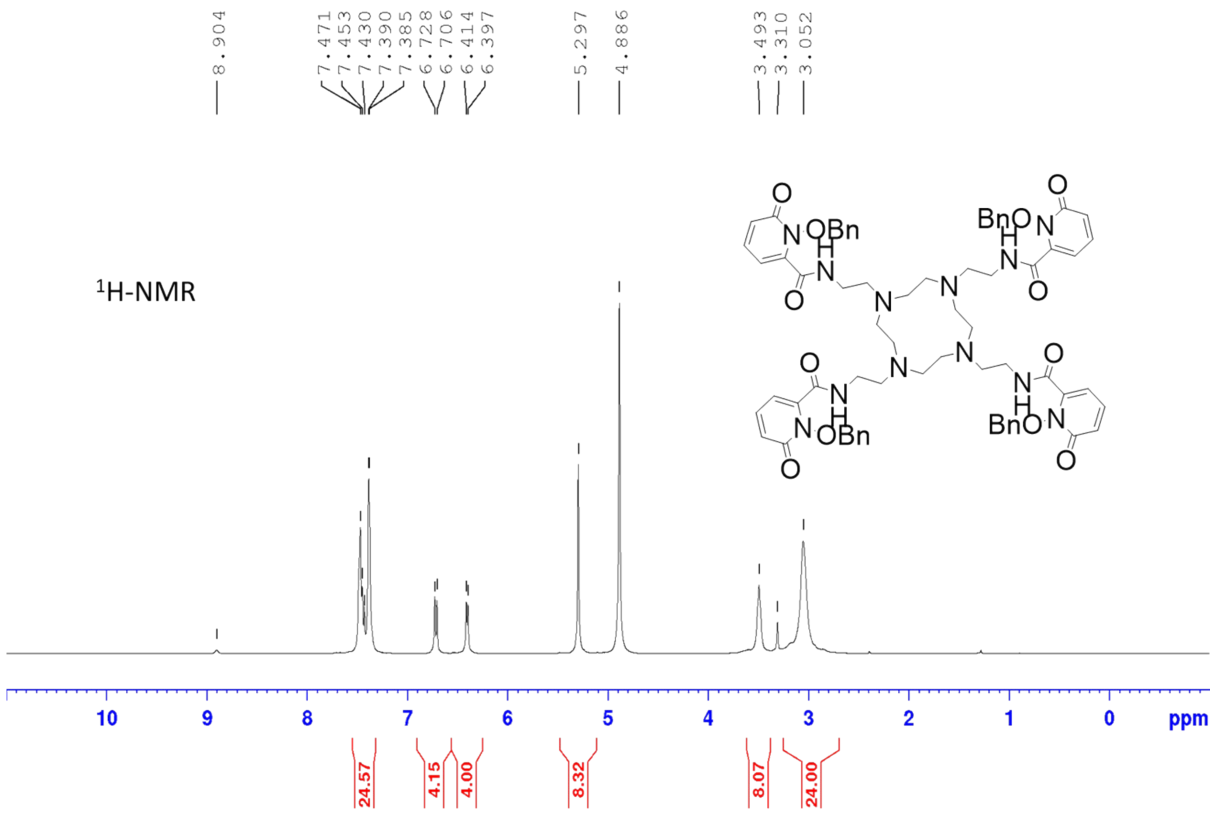

Figure S20. ^{1}H NMR spectrum of compound **3** in CD_3OD.

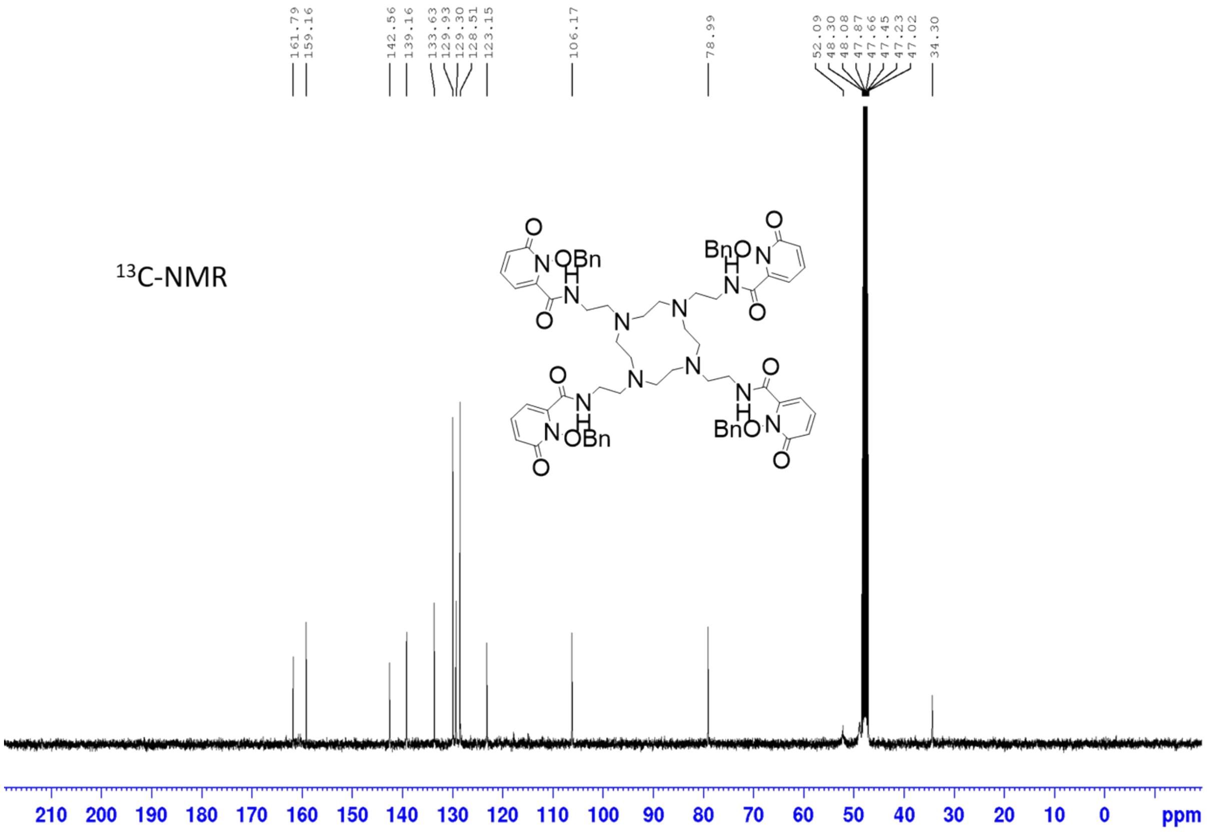

Figure S21. ^{13}C NMR spectrum of compound **3** in CD_3OD.

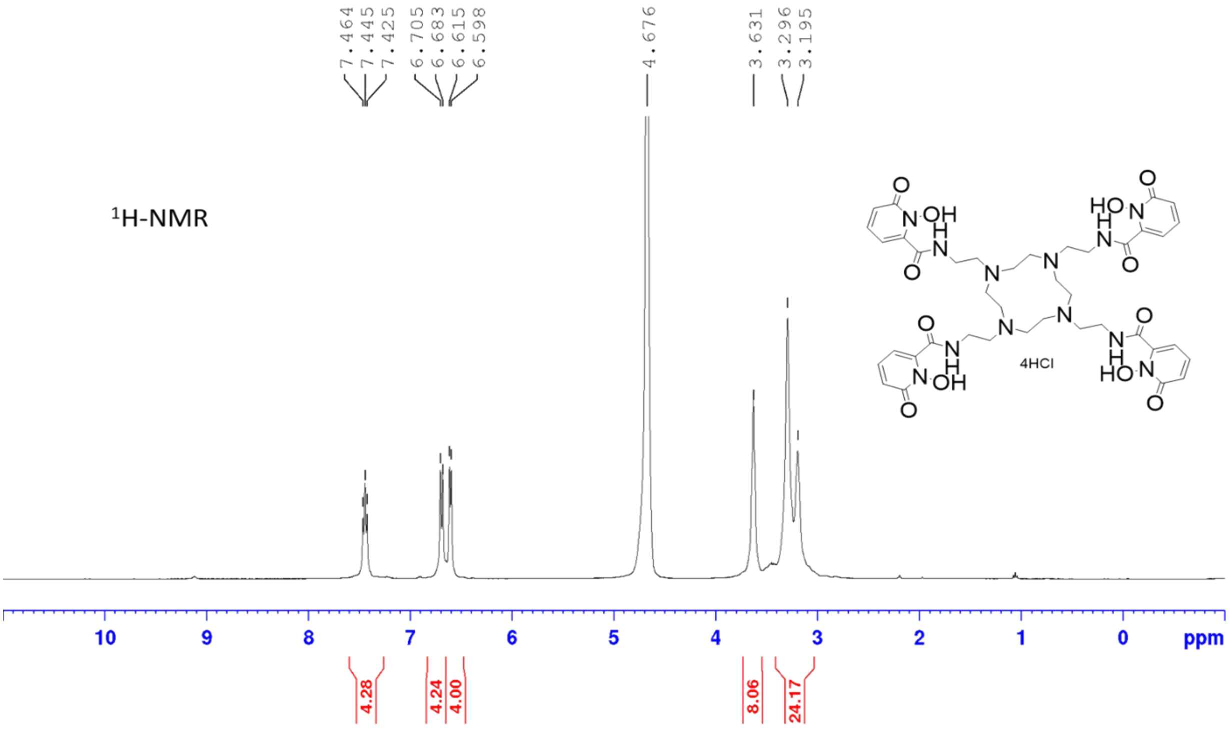

Figure S22. ^{1}H NMR spectrum of compound **4** in D_2O.

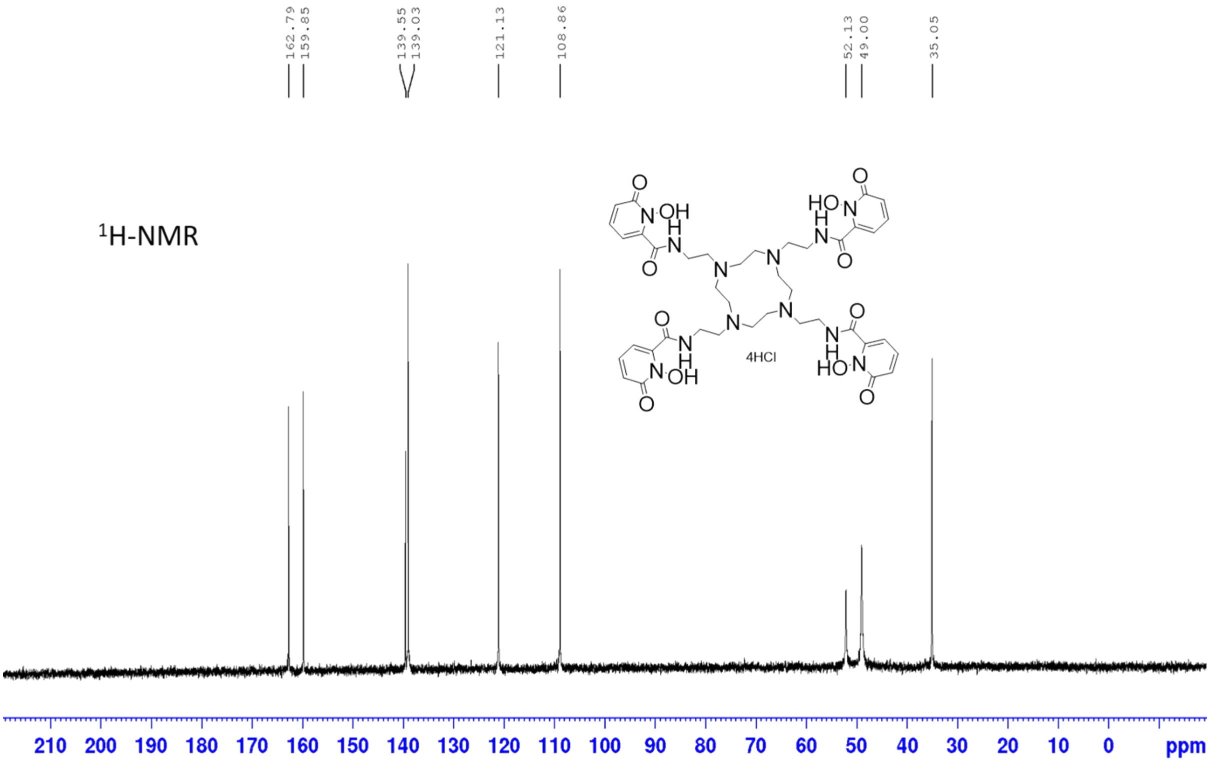

Figure S23. [1]C NMR spectrum of compound **4** in D_2O.

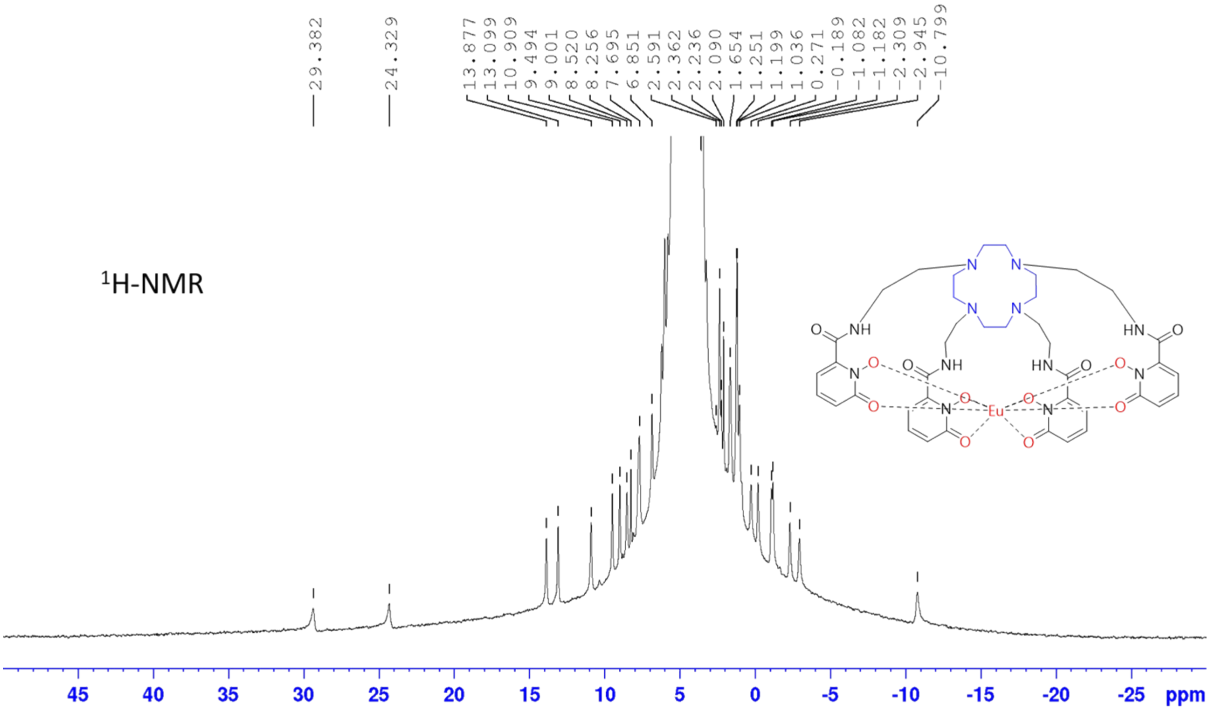

Figure S24. ^{1}H NMR spectrum of complex **Eu-Cy-HOPO** in D_2O.

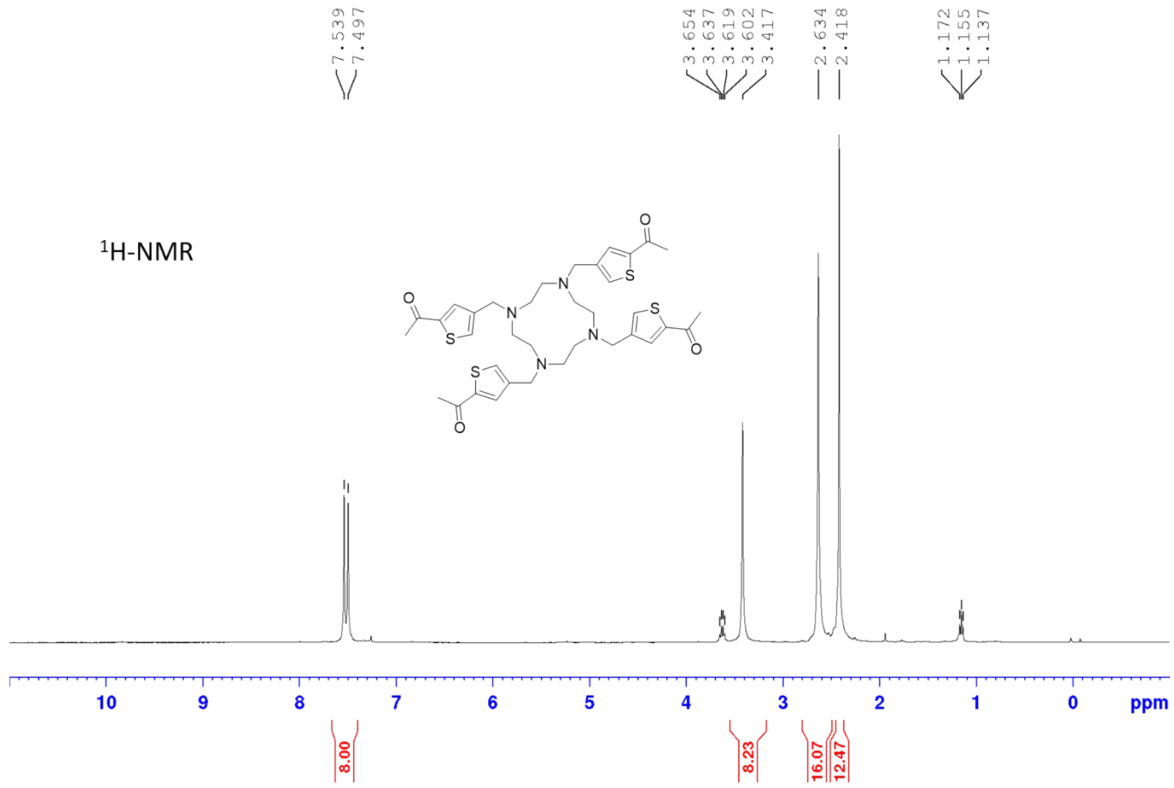

Figure S25. ^{1}H NMR spectrum of compound **5** in $CDCl_3$.

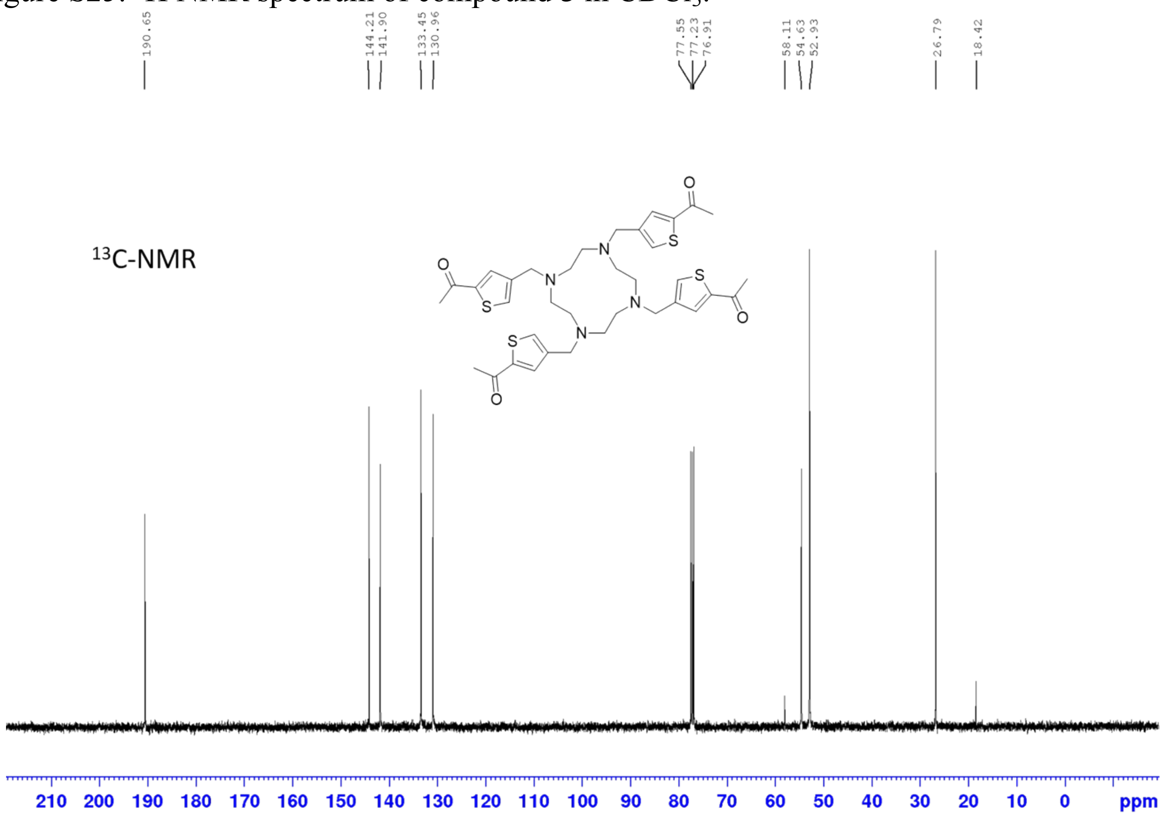

Figure S26. ^{13}C NMR spectrum of compound **5** in $CDCl_3$.

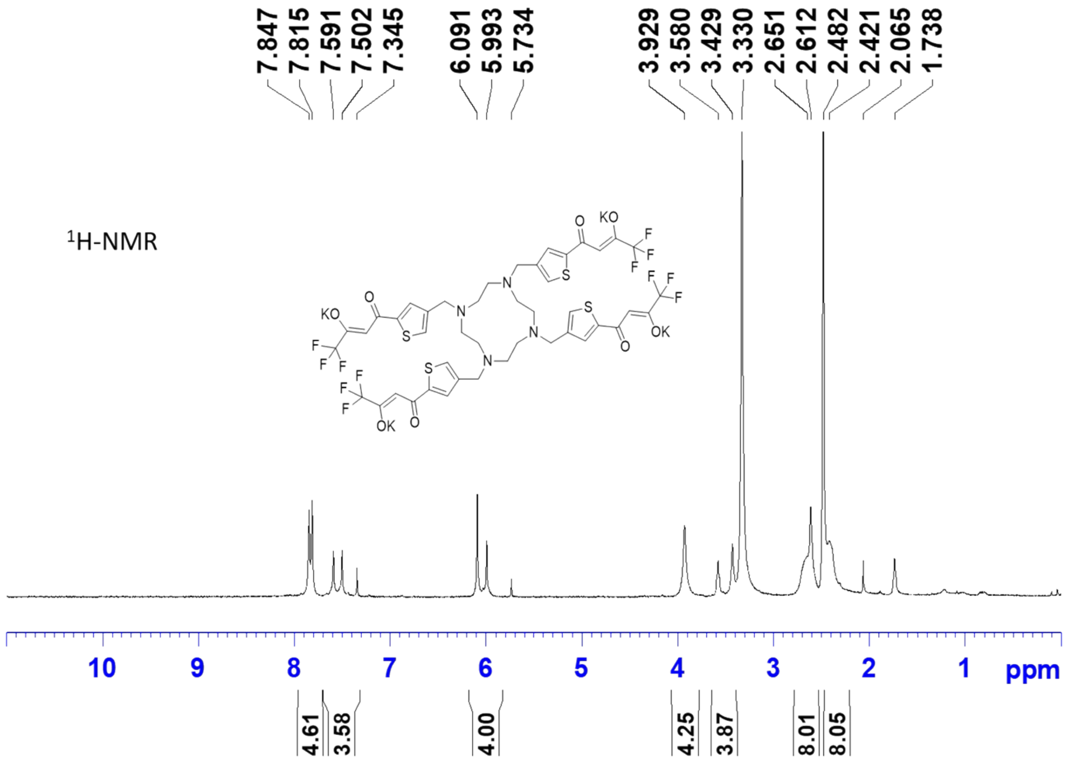

Figure S27. ^{1}H NMR spectrum of compound **6** in d[6]-DMSO.

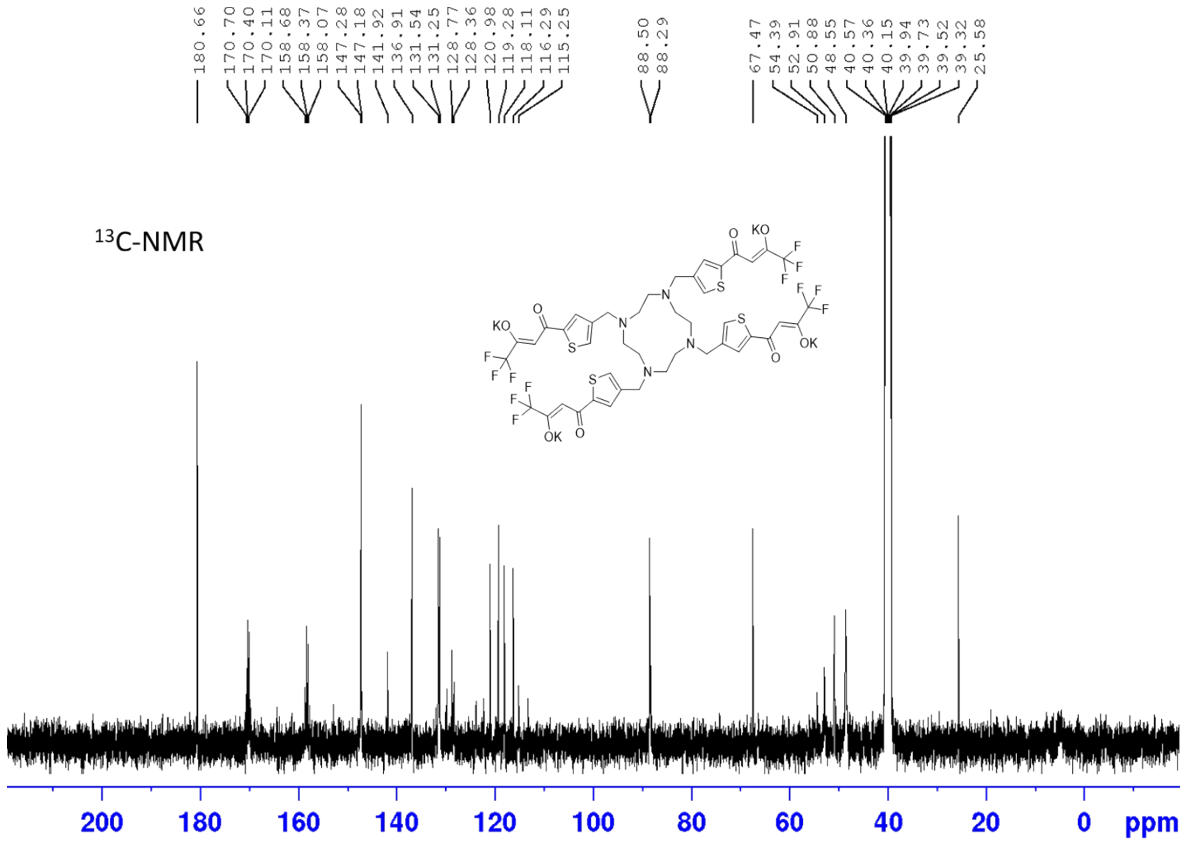

Figure S28. ^{13}C NMR spectrum of compound **6** in d[6]-DMSO.

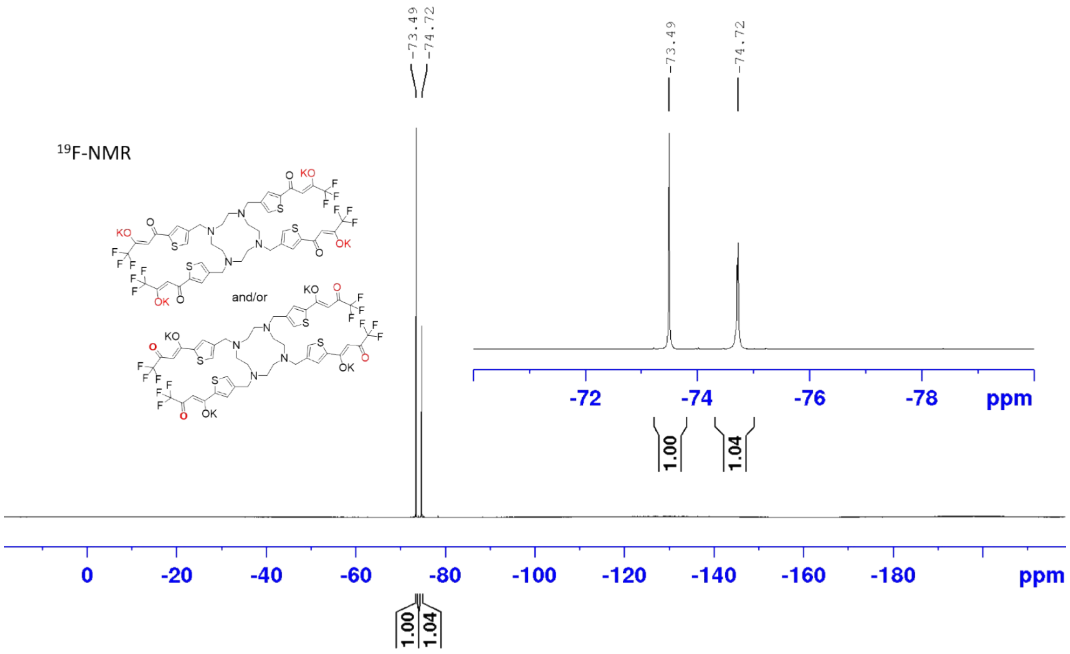

Figure S29. ^{19}F NMR spectrum of compound **6** in d^6-DMSO.

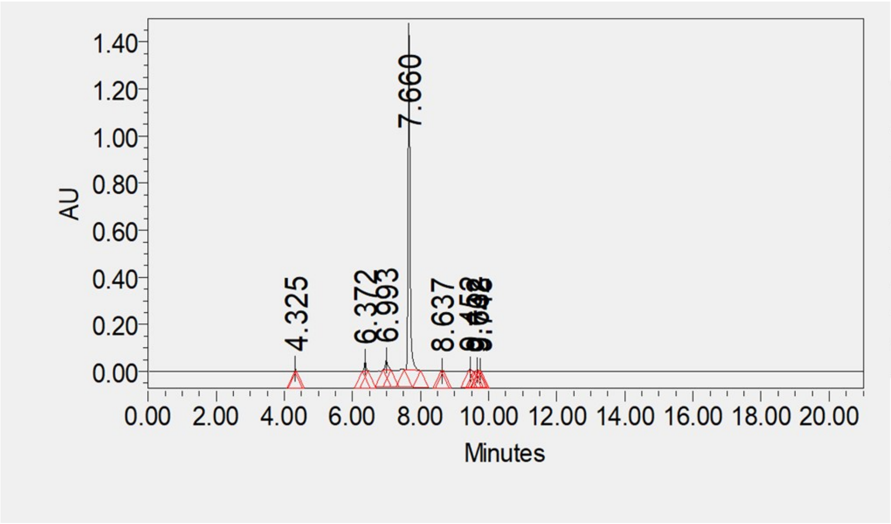

Retention Time (min)	% Area
4.325	0.21
6.372	2.27
6.993	3.38
7.660	93.60
8.637	0.07
9.452	0.29
9.662	0.15
9.748	0.03

Figure S30. RP-HPLC trace of complex **Eu-Cy-HOPO** (350 nm)

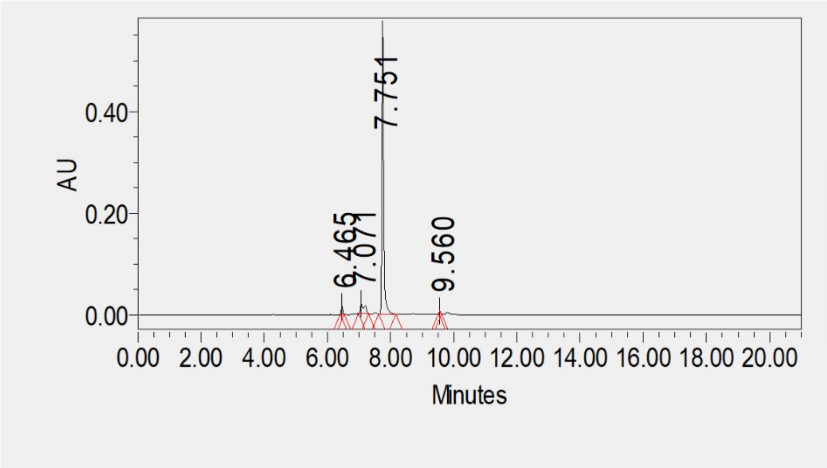

Retention Time (min)	% Area
6.465	2.14
7.071	7.53
7.751	89.64
9.560	0.68

Figure S31. RP-HPLC trace of complex **Sm-Cy-HOPO** (350 nm).

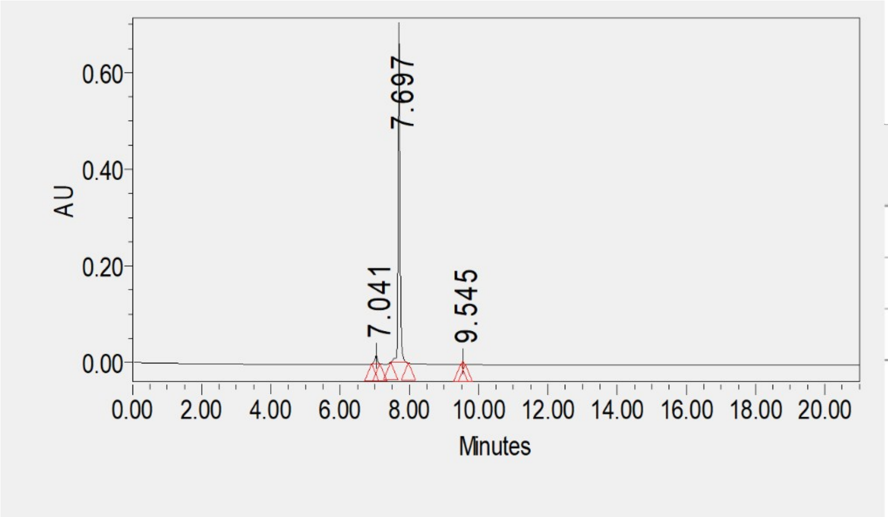

Retention Time (min)	% Area
7.041	3.26
7.697	95.97
9.545	0.76

Figure S32. RP-HPLC trace of complex **Gd-Cy-HOPO** (350 nm).

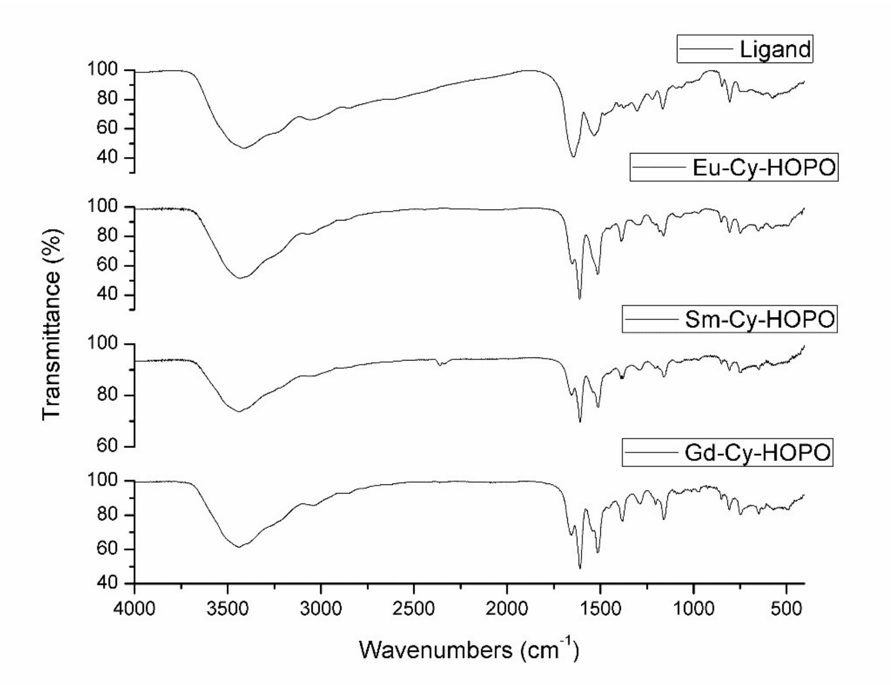

Figure S33. Comparison of IR spectra of ligand **4** and complexes of **Eu-Cy-HOPO**, **Sm-Cy-HOPO** and **Gd-Cy-HOPO**.

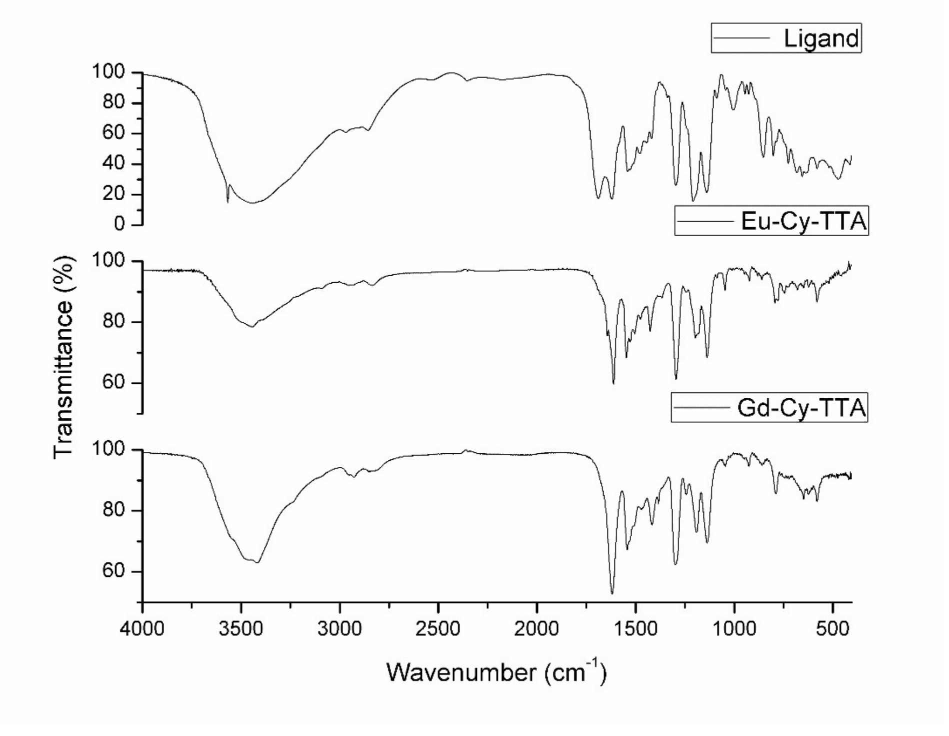

Figure S34. Comparison of IR spectra of ligand **6** and complexes of **Eu-Cy-TTA** and **Gd-Cy-TTA**.

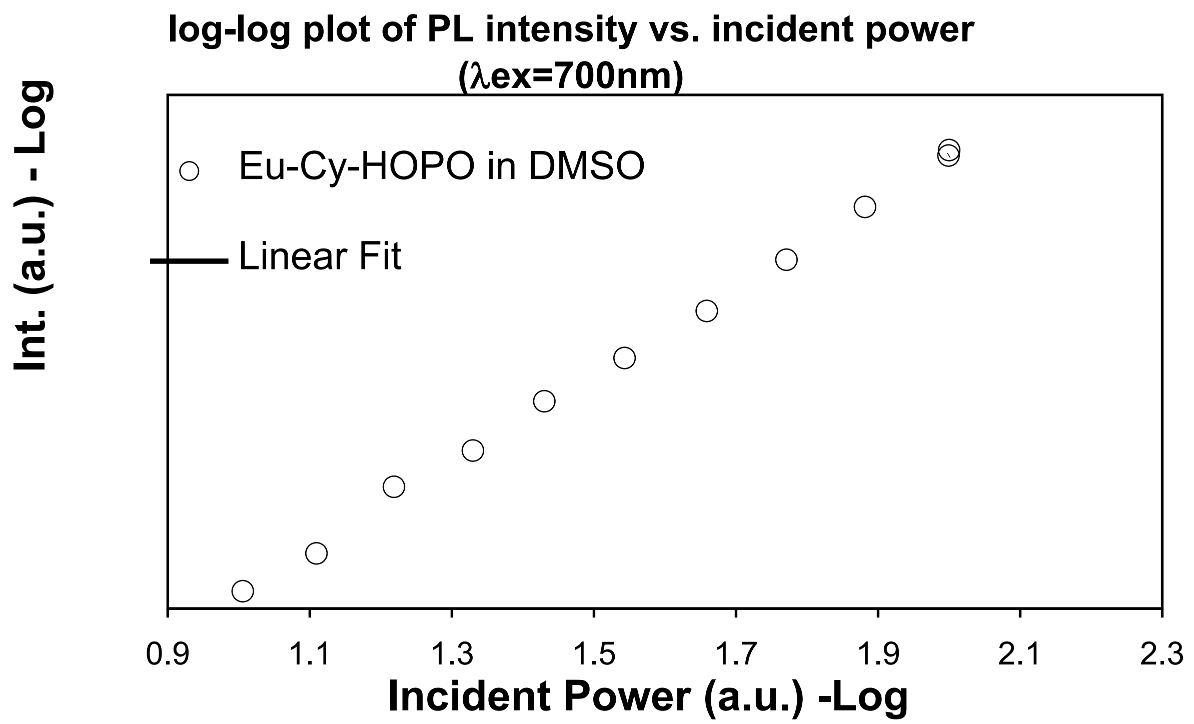

Figure S35. Dependence of luminescence intensity on incident power of **Eu-Cy-HOPO** in DMSO.

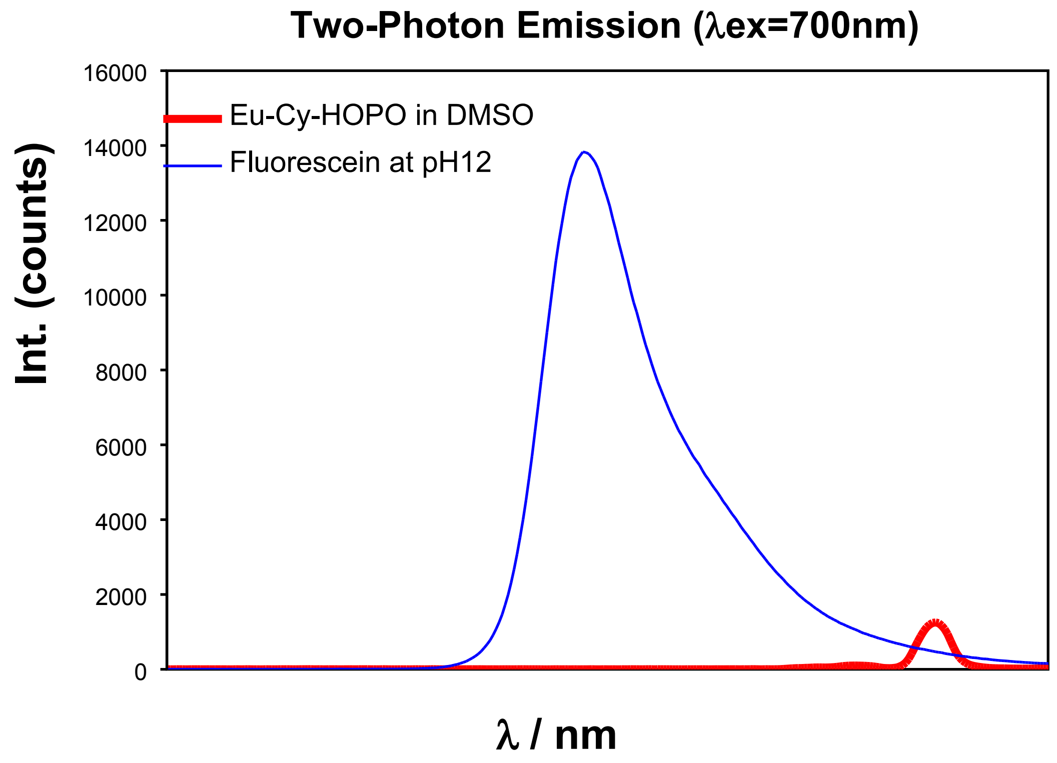

Figure S36. Two-photon excitation emission spectra of Fluorescein at pH 12 and **Eu-Cy-HOPO** in DMSO.

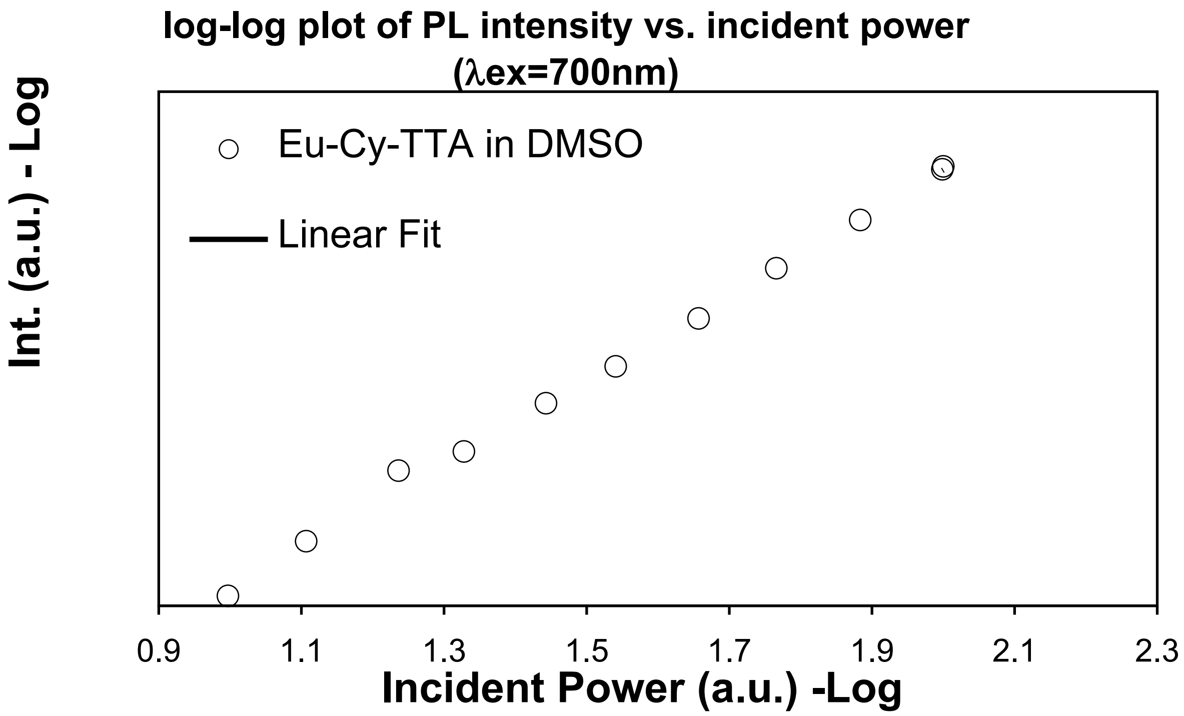

Figure S37. Dependence of luminescence intensity on incident power of **Eu-Cy-TTA** in DMSO.

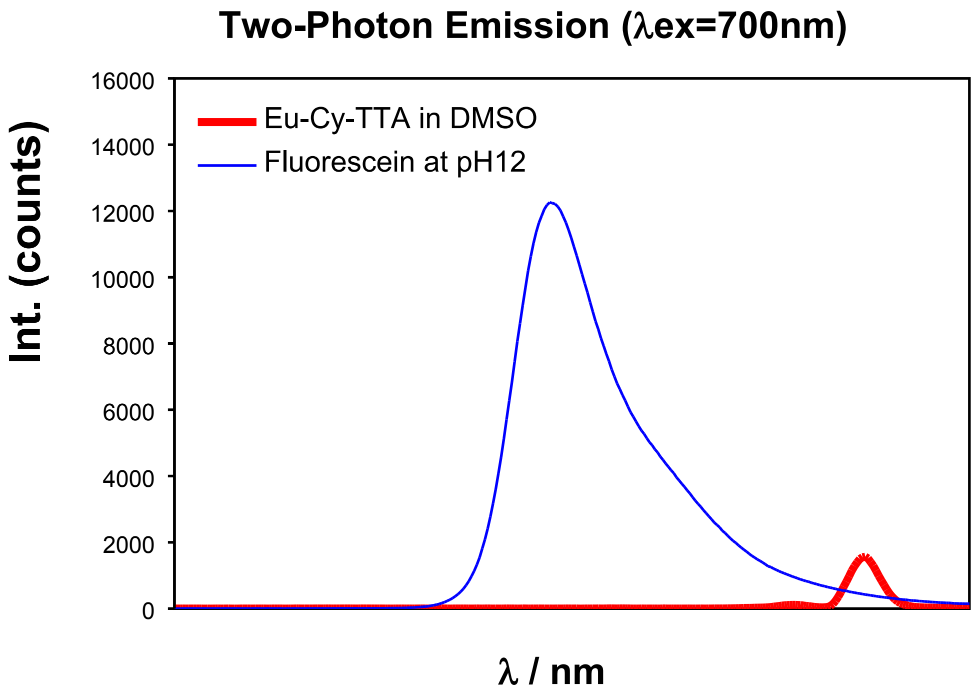

Figure S38. Two-photon excitation emission spectra of Fluorescein at pH 12 and **Eu-Cy-TTA** in DMSO.

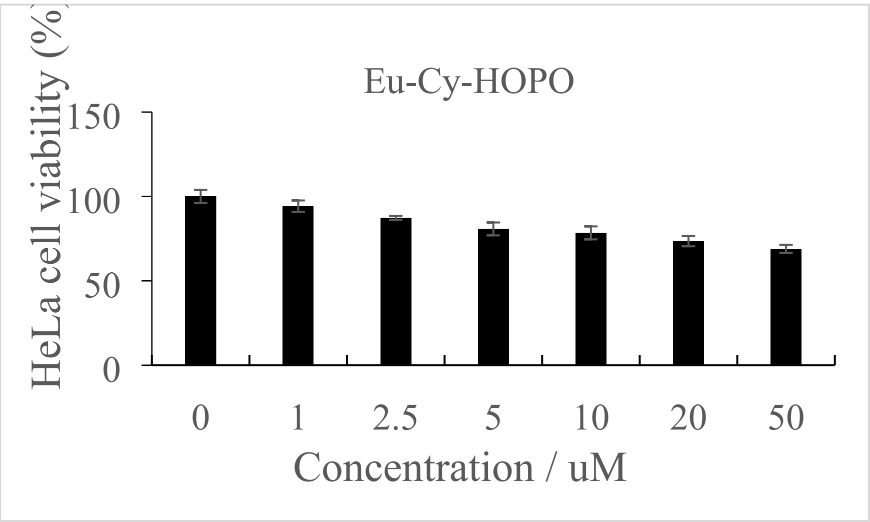

Figure S39. Viability of HeLa cells incubated with **Eu-Cy-HOPO** for 24 hours.

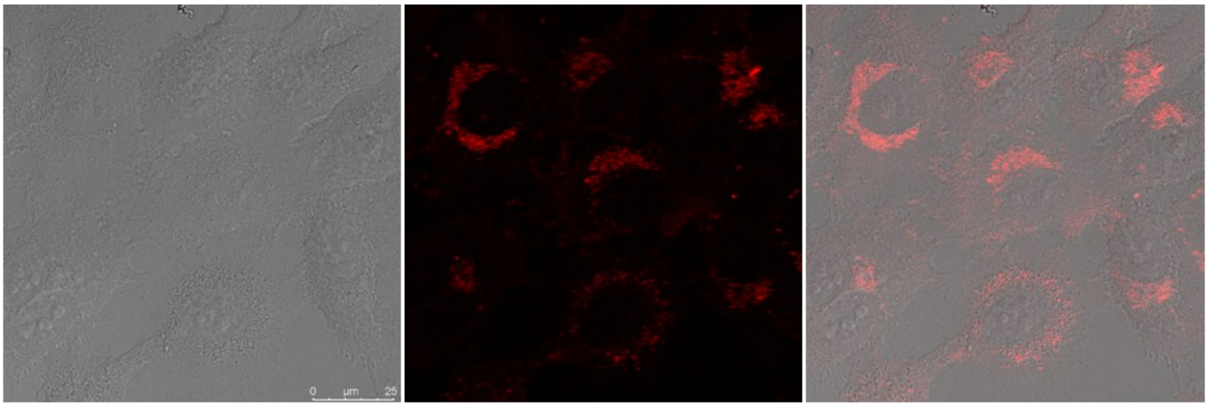

Figure S40. Bright field (left) and fluorescent microscopy image (middle) and overlaid image of **Sm-Cy-HOPO** (40 µM) in HeLa cells after 3 hours of incubation.

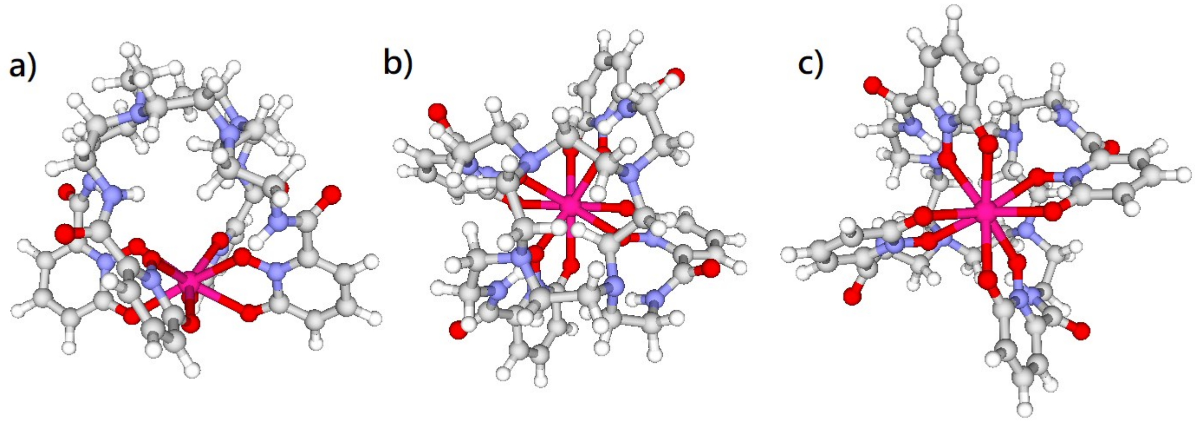

Figure S41. Optimized structure of **Sm-Cy-HOPO**. View from side (a); above cyclen backbone (b); view from below Sm(III) center (c).

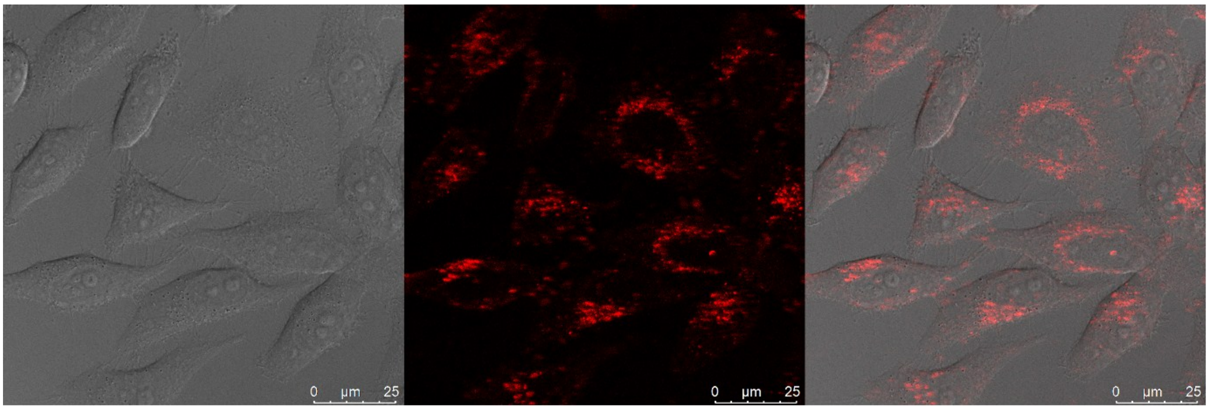

Figure S42. Bright field (left) and multi-photon microscopy image (middle) and overlaid image (right) of **Eu-Cy-HOPO** (4 µM) after 3 hours of incubation (λ_{ex} = 760 nm).
